# The amplitude in periodic neural state trajectories underlies the tempo of rhythmic tapping

**DOI:** 10.1371/journal.pbio.3000054

**Published:** 2019-04-08

**Authors:** Jorge Gámez, Germán Mendoza, Luis Prado, Abraham Betancourt, Hugo Merchant

**Affiliations:** Instituto de Neurobiología, Universidad Nacional Autónoma de México, Campus Juriquilla, Querétaro, México; McGill University, CANADA

## Abstract

Our motor commands can be exquisitely timed according to the demands of the environment, and the ability to generate rhythms of different tempos is a hallmark of musical cognition. Yet, the neuronal underpinnings behind rhythmic tapping remain elusive. Here, we found that the activity of hundreds of primate medial premotor cortices (MPCs; pre-supplementary motor area [preSMA] and supplementary motor area [SMA]) neurons show a strong periodic pattern that becomes evident when their responses are projected into a state space using dimensionality reduction analysis. We show that different tapping tempos are encoded by circular trajectories that travelled at a constant speed but with different radii, and that this neuronal code is highly resilient to the number of participating neurons. Crucially, the changes in the amplitude of the oscillatory dynamics in neuronal state space are a signature of duration encoding during rhythmic timing, regardless of whether it is guided by an external metronome or is internally controlled and is not the result of repetitive motor commands. This dynamic state signal predicted the duration of the rhythmically produced intervals on a trial-by-trial basis. Furthermore, the increase in variability of the neural trajectories accounted for the scalar property, a hallmark feature of temporal processing across tasks and species. Finally, we found that the interval-dependent increments in the radius of periodic neural trajectories are the result of a larger number of neurons engaged in the production of longer intervals. Our results support the notion that rhythmic timing during tapping behaviors is encoded in the radial curvature of periodic MPC neural population trajectories.

## Abstract 2

Beat-based timing depends on the amplitude of periodic neural population dynamics and on the number of engaged neurons in primate medial premotor areas.

## Author summary

The ability to extract the regular pulse in music and to respond in synchrony to this pulse is called beat synchronization and is a natural human behavior exhibited during dancing and musical ensemble playing. A part of the brain called the medial premotor cortex has been associated with rhythmic entrainment, and yet the neural basis of this complex behavior is still far from known. In this work, we recorded the neuronal activity from the medial premotor cortices of macaques trained to tap rhythmically to the frequency of a metronome. Using principal component analysis, we projected the time-varying activity of hundreds of neurons into a low-dimensional space. The projected activity of the neural population generated a circular trajectory for every interval produced in the sequence, which travelled at a constant speed but with different radii for different tapping tempos. In addition, the increase in amplitude and variability of the neural trajectories accounted for the scalar property of timing, a generalized feature of temporal processing across tasks and species and which defines a linear relationship between the variability of temporal performance and interval duration.

## Introduction

Precise timing is a fundamental requisite for a select group of complex actions such as the execution and appreciation of music and dance [1]. In these behaviors, the perception of time intervals is facilitated by the presence of a regular beat in the rhythmic sequence, and individual intervals are encoded relative to this pulse or beat. This is called beat-based timing and serves as a framework for rhythmic entrainment, in which subjects perform movements synchronized to music [2–4]. Most of occidental music is organized by a quasi-isochronous pulse and frequently also in a metrical hierarchy, in which the beats of one level are typically spaced at two or three times those of a faster level (i.e., the tempo of one level is 1/2 [march meter] or 1/3 [waltz meter] that of the other), and humans can typically synchronize at more than one level of the metrical hierarchy [5,6]. Rhythmic tapping to an isochronous metronome is the simplest case of beat entrainment [7] and has been thoroughly studied in humans [8,9]. In contrast to the large human flexibility to perceive and entrain to complex beats in music, nonhuman primates can perceive [10–13] and synchronize to simple isochronous beats [14–16]. On the other hand, other sets of behaviors, such as the interception of a moving target or the production of a single interval, seem to depend on a duration-based timing mechanism, in which the absolute duration of individual time intervals is encoded discretely, like a stopwatch [2,17]. Functional imaging and behavioral studies have suggested the existence of a partially segregated timing neural substrate, with the cerebellum as a key structure for duration-based timing, the basal ganglia as main nuclei for beat-based timing, and medial premotor cortices (MPCs; which include the pre-supplementary motor area [preSMA] and supplementary motor area [SMA]) as a potential master clock for both timing mechanisms [7,18–20]. Yet, the neural substrate for absolute timing, and especially for beat perception and rhythmic entrainment, is still largely unknown.

Recent advances on the neurophysiology of absolute timing during single interval reproduction tasks suggest that time is represented in the structured patterns of activation of cell populations in timing areas such as the MPC and the neostriatum [21–24]. Rather than being quantified in the instantaneous activity of single cells that accumulate elapsed time or encode the time remaining for an action [25–27], the duration of produced intervals depends on the speed at which the neural population response changes. This implies that the activation profiles are compressed for short and elongated for long intervals due to temporal scaling on the activity of the same population of cells [23,24].

On the other hand, MPC neurons are tuned to the duration and ordinal sequence of rhythmic movements produced either in synchrony with a metronome or guided by an endogenous tempo (synchronization-continuation task [SCT]) [4,21]. Remarkably, the time-varying profile of activation of these interval-specific neural circuits forms a moving bump, which is defined as a sequential pattern of responses in which the cells are activated consecutively within a produced interval. The moving bump repeats itself on each produced interval of the tapping sequence [4,21,28]. Nevertheless, single MPC cells multiplex the interval, the serial order, and task phase of the SCT, showing complex and heterogenous time-varying profiles of activation that make it difficult to understand the neural population mechanisms behind rhythmic tapping. A successful approach to determine the latent task variables in cell populations is to project high-dimensional individual neural activity into a low-dimensional topological space, in order to generate a robust and stable manifold [29]. Recent studies have reconstructed key hidden task parameters in the neural state population dynamics [30–32]. Thus, the combined use of high-density single unit recordings with dimensional reduction methods have revealed basic organizing principles at the level of the population dynamics, which seem to be extremely complex at the level of individual neurons [29,33].

Here, we investigated the population dynamics of hundreds of MPC neurons in monkeys performing two isochronous tapping tasks, testing whether low-dimensional state network trajectories can act as a neural clock during rhythmic tapping. Using dimensional reduction analysis, we found highly stereotyped neural trajectories that had two main properties during the SCT. First, the three first principal components showed a periodic path for each produced interval. Notably, these oscillatory state trajectories did not overlap across durations, a signature of temporal scaling; instead, they showed a linear increase in their radius and a constant linear speed as a function of the target interval during metronome guidance (synchronization condition [SC]), as well as during internally controlled rhythmic tapping (continuation condition [CC]). Second, the intertrial variability of the trajectories’ radial magnitude also increased as a function of the interval, accounting for a key feature of timing behavior: the scalar property, which states that the variability of produced or estimated intervals increases linearly as a function of interval duration. These properties were highly resilient to the number of participating neurons and were replicated using simultaneously recorded cells during synchronized tapping, but not during a serial reaction time-control task that precluded rhythmic prediction. Finally, we found a tight correlation between the interval-associated changes in trajectory amplitude and variability during SCT, the number of neurons involved in the sequential transient activation patterns, and the duration of the neural activation periods within these moving bumps. Indeed, moving bumps simulations revealed that scaling the duration of the transient period of activity and increasing the number of neurons participating in the evolving patterns produced an increase in the radius and the variability of the corresponding neural trajectories, replicating the empirical findings. These results suggest that rhythmic timing depends on the radial amplitude of periodic state population trajectories in MPC, which in turn depend on the number of neurons involved and the duration of these cells’ activation periods within moving bumps.

## Results

### Rhythmic tapping behavior

**Fig 1:**
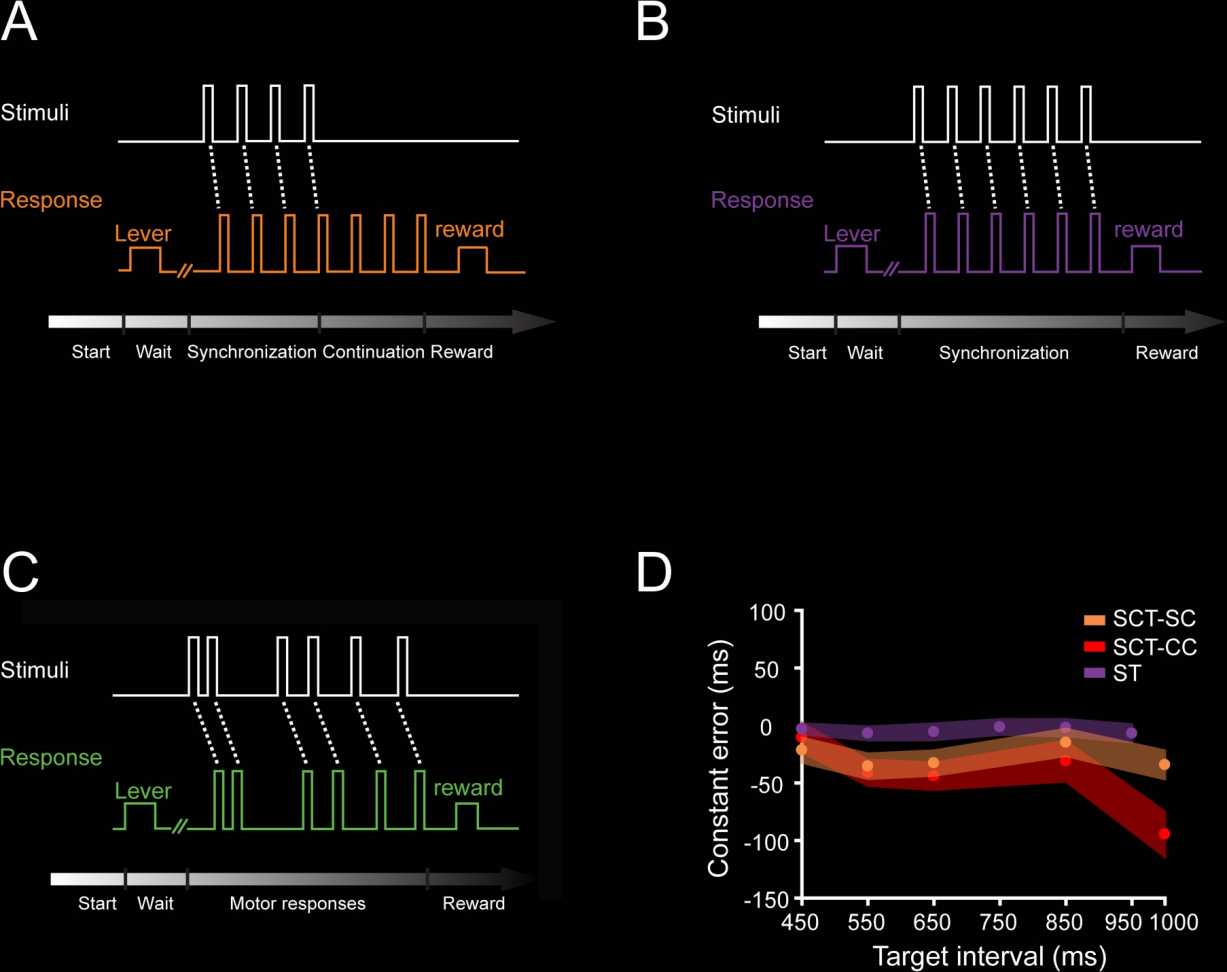
Tasks. **A**. SCT. The trial started when the monkey placed his hand on a lever for a variable delay. Then, a visual metronome was presented, and the monkey tapped on a button to produce three intervals of a specific duration following the isochronous stimuli (synchronization phase), after which the animal had to maintain the tapping rate to produce three additional intervals without the metronome (continuation phase). Correct trials were rewarded with an amount of juice that was proportional to the trial length. The instructed target intervals were 450, 550, 650, 850, and 1,000 ms. **B**. ST. Similar to the synchronization phase of the SCT, the animal had to produce five intervals guided by a visual metronome. The instructed intervals were 450, 550, 650, 750, 850, and 950 ms. **C**. SRTT. As in ST, the trial started when the monkey placed its hand on a lever for a variable delay. However, in this task, the monkey tapped the button after six stimuli separated by a random interstimulus interval, precluding the temporalization of the tapping behavior. **D.** Constant error (mean ± SD/2) as a function of target interval during the SC (orange) and CC (red) of the SCT (ANOVA main effect interval, F(4, 1,112) = 61.01, *p* < 0.0001; main effect task condition, F(1, 1,112) = 43.16, *p* < 0.0001; interval × condition interaction, F(4, 1,112) = 17.66, *p* < 0.0001), and the ST (purple) as a function of target interval (ANOVA for 450, 550, 650, and 850 target intervals between SC of the SCT and the ST, main effect interval, F(3, 631) = 4.18, *p* < 0.01; main effect condition, F(1, 631) = 202.16, *p* < 0.0001; nonsignificant interval × condition interaction, F(3, 631) = 2.46, *p* = 0.06). Underlying data are available in https://doid.gin.g-node.org/d315b3db0cee15869b3d9ed164f88cfa/. CC, continuation condition; SC, synchronization condition; SCT, synchronization-continuation task; SRTT, serial reaction time task; ST, synchronization task.

**Fig 2:**
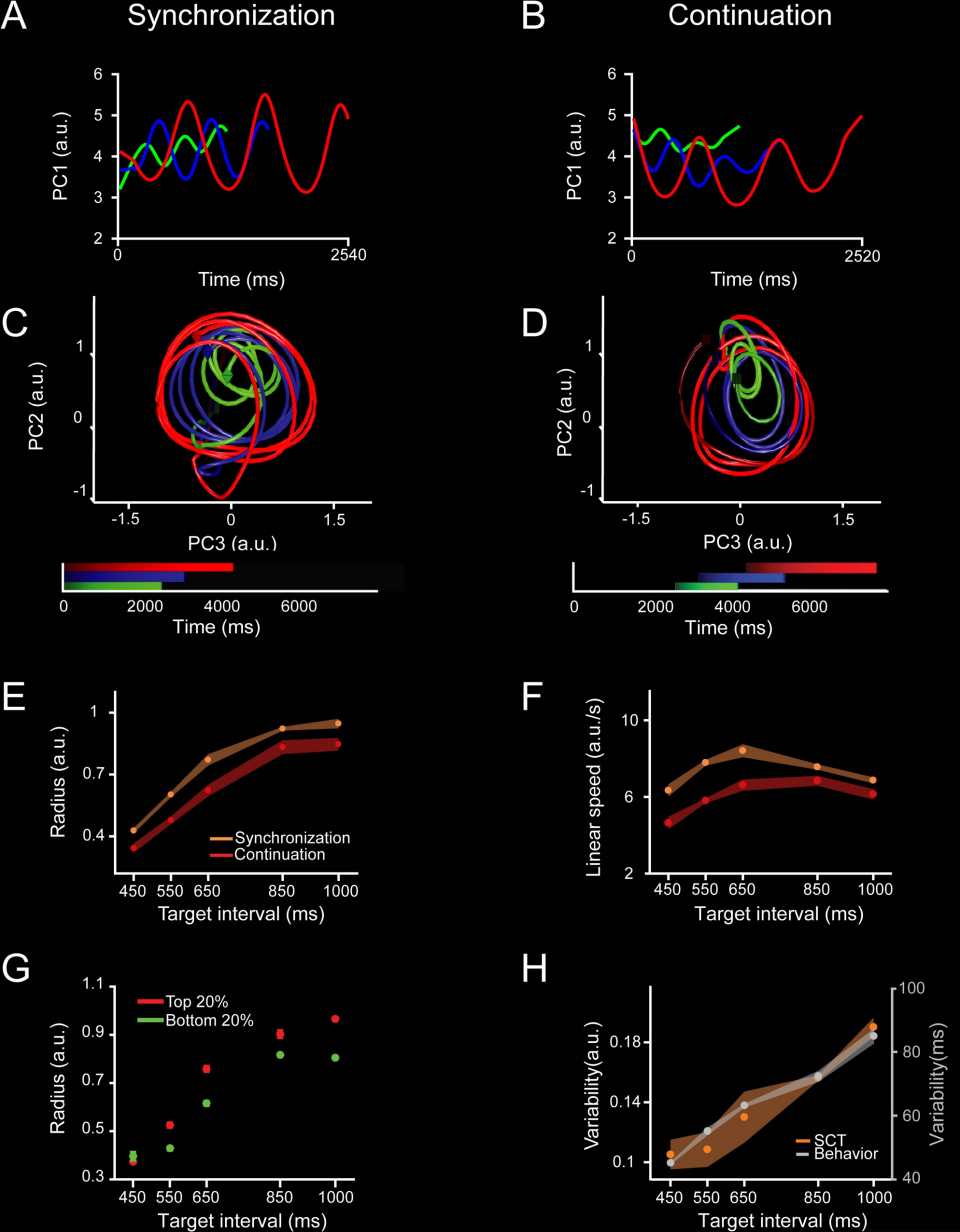
Neural population trajectories during SCT and their oscillatory dynamic properties. **A, C**. Projection of the neural activity in the MPC (1,477 neurons) during the SC of the SCT onto the first (**A**) or second and third PCs (**C**). The first three PCs explained the 10.7%, 3.8%, and 2.3% of the total variance. Each point in the trajectory represents the neural network state at a particular moment. The trajectory completes an oscillatory cycle on every produced interval during the synchronization and continuation phases of the SCT. Target interval in milliseconds is color coded (450, green; 650, blue; 1,000, red). Color progression within each target interval corresponds to the elapsed time. A cube indicates the beginning of each trajectory, while an octahedron indicates the end. **B, D**. Projection of the neural activity during CC of the SCT onto the first (**B**) or the second and third (**D**) PC. Color code is the same as (**A**). **E**. Monotonic increase of the radii in the oscillatory neural trajectories during SC (orange, mean ± SD, slope = 0.0009, constant = 0.0679, *R*^2^ = 0.9, *p* = 0.01) and CC (red, mean ± SD, slope = 0.0009, constant = −0.0296, *R*^2^ = 0.9, *p* < 0.01) as a function of target interval. **F**. Linear speed of neural trajectories during SC (orange, mean ± SD, slope = 0.0001, constant = 7.322, *R*^2^ = 0.0007, *p* = 0.896) and CC (red, mean ± SD, slope = 0.002, constant = 4.049, *R*^2^ = 0.354, *p* = 0.002) as a function of target interval (ANOVA main effect interval, F(4, 39) = 92.15, *p* < 0.0001; main effect condition, F(1,39) = 381.46, *p* < 0.0001; interval × condition interaction, F(4, 39) = 15.15, *p* < 0.0001). The linear speed was similar (SC) or showed a slight increase (CC) with the target interval. **G.** Neural trajectory radii for the top 20% (red, slope = 0.0011, constant = −0.035, *R*^2^ = 0.7, *p* < 0.0001) and bottom 20% (green, slope = 0.00088, constant = −0.009, *R*^2^ = 0.75, *p* < 0.0001) inter-tap intervals across target intervals. Note that on those intervals in which the monkeys tended to produce shorter inter-tap durations, the state trajectory radius was smaller, and vice versa (ANOVA main effect interval, F(4, 40) = 155.7, *p* < 0.0001; main effect population, F(1, 40) = 33.3, *p* < 0.0001; interval × population interaction, F(4, 40) = 3.98, *p* = 0.008). **H**. Variability (SD) of SCT rotational neural trajectories (orange, mean ± SD, normalized data slope = 0.0019, constant = −1.02, *R*^2^ = 0.94, *p* = 0.005) and the monkeys’ produced intervals (gray, mean ± SD, normalized data slope = 0.005, constant = −0.721, *R*^2^ = 0.98, *p* = 0.0008) as a function of target interval. The Weber increase in tapping variability was not statistically different from the increase in the variability of neural trajectories across target intervals (normalized data, slope *t* test = 0.86, *p* = 0.42; constant *t* test = 1.36, *p* = 0.22). Underlying data are available in https://doid.gin.g-node.org/d315b3db0cee15869b3d9ed164f88cfa/. a.u., arbitrary unit; CC, continuation condition; MPC, medial premotor cortex; PC, principal component; SC, synchronization condition; SCT, synchronization-continuation task.

We trained two monkeys (M01 and M02) in the SCT. M01 was also trained in two additional tapping tasks: the synchronization task (ST) and the serial reaction time task (SRTT). During SCT, the animals tapped on a push button in synchronization with a rhythmic metronome for four times, thus producing three intervals (SC phase), followed by three internally generated intervals (CC phase; Fig 1A). In the ST, the monkey produced five intervals guided by a metronome, similarly to the SC of SCT (Fig 1B). During the SRTT, the animal pressed the button in response to five brief visual stimuli presented in a sequence but separated by a random interstimulus interval, precluding the prediction of the next stimulus-response loop (Fig 1C). Thus, during SCT and ST, the animals entrained their rhythmic movements to a sensory metronome, while in the CC of SCT, this was done to an internal representation of the same rhythm. The asynchronies in the SC of SCT were (mean ± SD: 288.7 ± 70 ms). On the other hand, the SRTT involved similar stimuli, tapping behavior, and sequential structure, but no predictive rhythmic timing was possible. Expectedly, the reaction times were significantly larger in the SRTT than the asynchronies in the ST (mean ± SD: 263 ± 37 ms in the ST and 381 ± 46 ms in the SRTT; ANOVA main effect of task: F(1, 718) = 1443.93, *p* < 0.0001). The constant error, a measure of timing accuracy that corresponds to the difference between the produced and the instructed interval, was slightly negative during SCT and ST, indicating that the monkeys were able to properly produce the intervals with a small underestimation across target durations (Fig 1D). Finally, the temporal variability (a measure of timing precision) during the SCT and ST are depicted in Fig 2H and Fig 4E, respectively.

### Neural state trajectories

We characterized the dynamics of the evolving response patterns using the projection of the neural population time-varying activity onto a low-dimensional state space using principal component analysis (PCA) on a population of 1,477 MPC cells recorded during SCT (see Materials and methods, recording locations in S1 Fig). The results showed highly stereotyped trajectories with a strong periodicity in the first three principal components (PCs) (Fig 2A–2D). Indeed, PC2 and PC3 showed together a cyclic path for each produced interval (Fig 2C and 2D). Each loop in the trajectory corresponded to the periodic network state variation during the production of the rhythmic tapping sequence of the SCT. The circular trajectories in the plane exhibited the tendency to start at the same position in the phase-space after each tap, suggesting the existence of a movement-triggering point at a particular location in the population trajectory across durations (see below). Crucially, from this common phase-space location, longer intervals produced larger state trajectory loops, with a monotonic increase in the trajectory radius as a function of target interval during both the SC and CC (Fig 2E). However, the observed interval-dependent modulations in curvilinear amplitude were not accompanied by modulations of the linear speeds of the periodic neural trajectories, as these remained constant across durations (Fig 2F). The same properties were observed in PC1 and when the PCA is computed from a subpopulation of neurons whose activity was task related (see S2 Fig). Hence, contrary to a prototypical temporal scaling, in which there is a decrease in linear speed as a function of interval and similar trajectory paths and traversed distances for different durations [24,34], the present results show that rhythmic timing during the SCT is represented as an increase in curvature radii in the neural network state dynamics.

To test the relationship between the radius of the curvature in the neural-state trajectories and the monkeys’ behavior during SC and CC, we split the produced intervals into two groups: those in which the monkeys produced an inter-tap time that was below the 20th percentile, and those with inter-tap times above the 80th percentile [21]. Strikingly, on those intervals in which the monkeys tended to produce shorter inter-tap durations, the state trajectory radius was smaller, and vice versa (Fig 2G).

Another important property of the curvilinear radii in the PCA neural trajectories was that their variability (SD of the trajectory radii) followed the same linear increase as a function of target interval observed in the monkeys’ behavior (Fig 2H). This linear relation between temporal variability and interval duration, known as scalar property of interval timing, has been widely reported in the timing literature, and our findings suggest that it depends on the radius of the rotatory dynamical state of MPC neural populations during both SCT conditions. It is important to mention that all the described properties in the neural trajectories are resilient on the methods used to compute the PCs (see S3 Fig).

**Fig 3:**
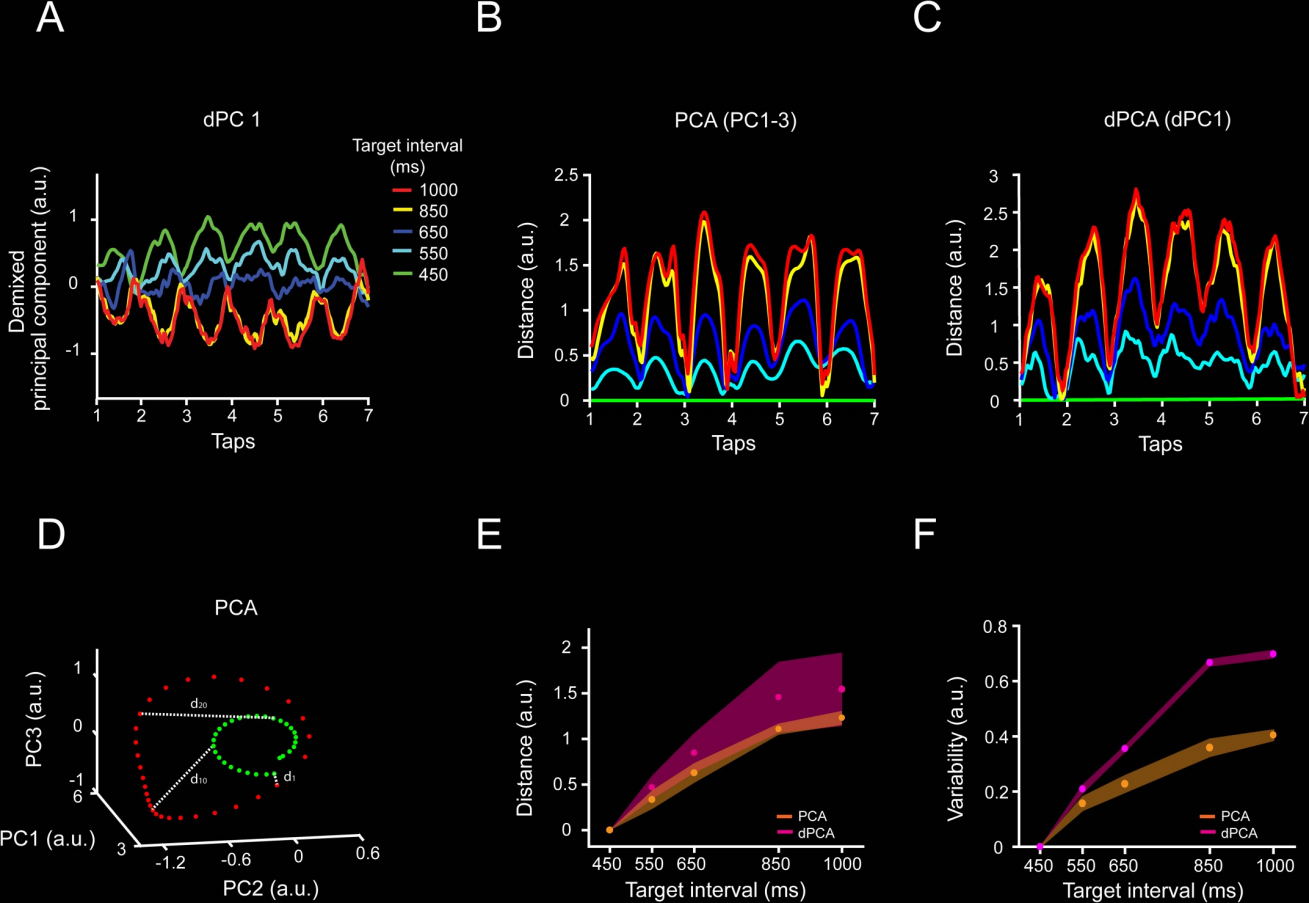
dPCA applied to neural population activity during SCT. **A,** dPC1 of the dPCA of the neural activity associated with the target interval (explains 7.8% of the total variance). Target interval in milliseconds is color coded (see inset **A**). The neural trajectories show oscillatory activity, and their amplitude varies across target intervals. **B,C.** Euclidean distance between the first PC of the 450-ms target interval and the first PC of each target interval across time for (**B**) time-normalized PCA and (**C**) dPCA. Target interval is color coded as in (**A**). Two-sample Kolmogorov–Smirnov test on the distributions of PCA and dPCA distances showed nonsignificant differences (*p* < 0.05) across target intervals. **D.** Distance calculation diagram for PCA data. The inter-tap trajectories for two target intervals are shown (green, 450 ms; red, 1,000 ms). The 450-ms target interval trajectory is used as the reference for distance calculation. The Euclidean distance between each sequential bin is calculated among the reference interval and the other target intervals trajectories. Both population analyses, PCA and dPCA, produced population signals with similar characteristics. Thus, oscillatory activity, modulation of the amplitude with the target interval, and an intersection close to the tap time are characteristics of the underlying neural population activity, irrespective of the dimension reduction algorithm. **E.** Mean inter-tap Euclidean distance (mean ± SD) between the 450-ms and each target interval for the PCA data using PC1–3, (orange) and dPCA using dPC1 (magenta). There was no significant difference between the slopes of PCA and dPCA (slope *t* test = 1.97, *p* = 0.0539) **F.** Variability of the distance between the 450-ms and each target interval for the PCA (orange) and dPCA (magenta). The variability increased monotonically as a function of the target interval for both analyses. Underlying data are available in https://doid.gin.g-node.org/d315b3db0cee15869b3d9ed164f88cfa/. a.u., arbitrary unit; dPCA, demixed PCA; PC, principal component; PCA, principal component analysis; SCT, synchronization-continuation task.

The dynamics in the MPC population activity during the SCT was also characterized using demixed PCA (dPCA; Fig 3, see Materials and methods). This method not only captures most of the variance in the neural data but, most importantly, also decomposes the dependencies of the neural population activity into latent components associated with task parameters [30]. In contrast, PCA only focuses on the total variance explained using orthogonal decomposition. The first dPCA (dPCA1) showed a strong periodic structure with a minimum value around the beginning of each produced interval in the SCT sequence, similar to the findings from the PCA neural trajectories (Fig 2C and 2D). In addition, the dPCA1 showed a strong change in amplitude with target duration (Fig 3A). Because we used time-normalized neural data as input to the dPCA, all trials had the same length regardless of the target interval. In this scenario, a scaling mechanism should have produced similar dPCAs across durations. Instead, we observed a time-dependent modulation in dPCA1 amplitude. In order to compare the two methods for dimensional reduction, we computed the bin-by-bin distance between the 450-ms and the other four target intervals (Fig 3D) using the PCAs (Fig 3B) and dPCA1 (Fig 3C). The resulting distance profiles are very similar between methods, with a periodic structure whose amplitude mean and variability increased as a function of the target interval (Fig 3E and 3F). Thus, with a separate set of assumptions, the dPCA corroborates the existence of both the periodic structure of the neural state dynamics and a beat-based timing mechanism based on the amplitude modulation of the rotatory population trajectories during SCT.

**Fig 4:**
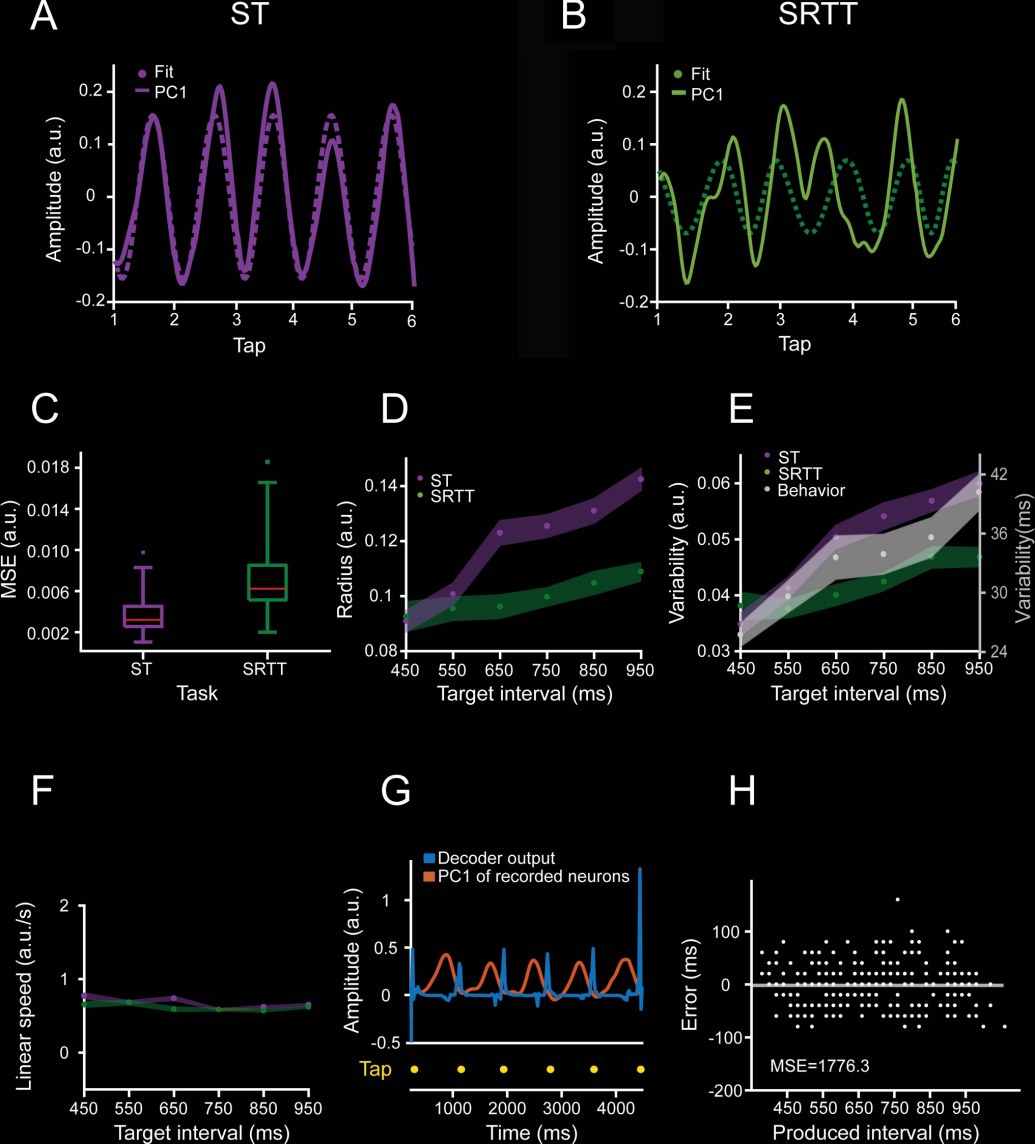
Comparison of ST and SRTT trajectories in simultaneously recorded neurons. **A**. Neural activity data projected on the PC1 (solid line, linearly detrended) and the correspondent sinusoidal fit (dotted line) during a trial of ST for the target interval of 650 ms. **B**. Similar to **(A)** for SRTT. Note that the strong periodic structure of the ST neural trajectory is lost during SRTT for the same population of cells. **C**. The MSE of the sinusoidal fits during ST (purple) is significantly smaller than during SRTT (green; 60 trials, two-sample *t* test = −6.78, *p* < 0.0001). **D**. Radii of the neural trajectories during ST (purple, slope = 0.000087, constant = 0.055, *R*^2^ = 0.619, *p* < 0.0001) and SRTT (green, nonsignificant linear regression, *R*^2^ = 0.0172 and *p* = 0.489) as a function of target interval. **E.** Variability of the neural trajectories during ST (purple, data slope = 0.000037, constant = 0.028, *R*^2^ = 0.368, *p* < 0.0001), SRTT (green, nonsignificant linear regression, *R*^2^ = 0.0005 and *p* = 0.903), and temporal variability of the monkeys’ produced intervals (gray, mean ± SD/2, data slope = 0.0009, constant = −0.003, *R*^2^ = 0.999, *p* < 0.0001) across target intervals during ST. **F.** Linear speed of neural trajectories during ST (purple, mean ± SD, slope = 0.0001, constant = 7.322, *R*^2^ = 0.0007, *p* = 0.896) and SRTT (green, mean ± SD, slope = 0.002, constant = 4.049, *R*^2^ = 0.354, *p* = 0.002) did not change as a function of target interval. **G.** Output of the time-delay neural network (TDNN, in blue) trained to decode the duration of produced intervals based on the PC1 neural trajectories (orange) during a target interval of 850 ms. Tapping times are shown in yellow. **H.** TDNN error, defined as the difference between the produced and the decoded interval, as a function of produced interval. TDNN predicted accurately the performance of the monkey on a trial-by-trial basis (the decoded mean was not statistically different from 0, *t* test = −0.5228, *p* = 0.6). Underlying data are available in https://doid.gin.g-node.org/d315b3db0cee15869b3d9ed164f88cfa/. a.u., arbitrary unit; MSE, mean square error; PC, principal component; SRTT, serial reaction time task; ST, synchronization task; TDNN, time-delay neural network.

The analyses described above were done on neurons recorded throughout different sessions. Thus, as a next step we determine the neural state trajectories on simultaneously recorded cells while monkey M01 performed an ST (Fig 1B) and an SRTT (Fig 1C). This strategy not only allows us to validate the data of the SCT on the ST but also permits us to determine population dynamics on a trial-by-trial basis. As in the SCT, the PCA-projected activity during the ST showed periodic state dynamics (Fig 4A; S4A Fig), whereas the SRTT neural trajectories were not as periodic (Fig 4B; S4B Fig). In fact, the fitting of a normalized sinusoidal function on the first PC was statistically more robust for ST than SRTT (in terms of mean square error [MSE]: Fig 4C), even when the length of the inter-tap PCA-projected activity was matched between different produced intervals (see Materials and methods). Again, the radius of the neural trajectories during the ST showed a significant increase in both mean radius (Fig 4D, purple) and variability (Fig 4E), but a constant linear speed (Fig 4F), as a function of the target interval, reproducing the findings in SCT. In contrast, the radius and variability of the trajectories during SRTT showed small changes across target intervals, with a nonsignificant linear fit as a function of target interval for the three parameters (Fig 4D, 4E and 4F, green). This phenomenological comparison suggests that rhythmic tapping to a metronome depends on the amplitude of the cyclic dynamics of population activity and that the shift from a predictive to a reactive behavior during SRTT precludes the organization of periodic population state trajectories.

The simultaneity of the recordings during ST [35] allowed for the decoding of the produced intervals on a trial-by-trial basis. Using a time-delay neural network (TDNN; see Materials and methods) (Fig 4G), we found that an ideal reader of the neural trajectories could predict accurately the tapping times during ST on 86% of the produced intervals. Indeed, the decoding accuracy was better than the actual percent of correct trials in this demanding task (Fig 4H), supporting the notion that the neural trajectories can robustly predict the rhythmic tapping behavior.

### The population state dynamics are not related to the tapping kinematics

**Fig 5:**
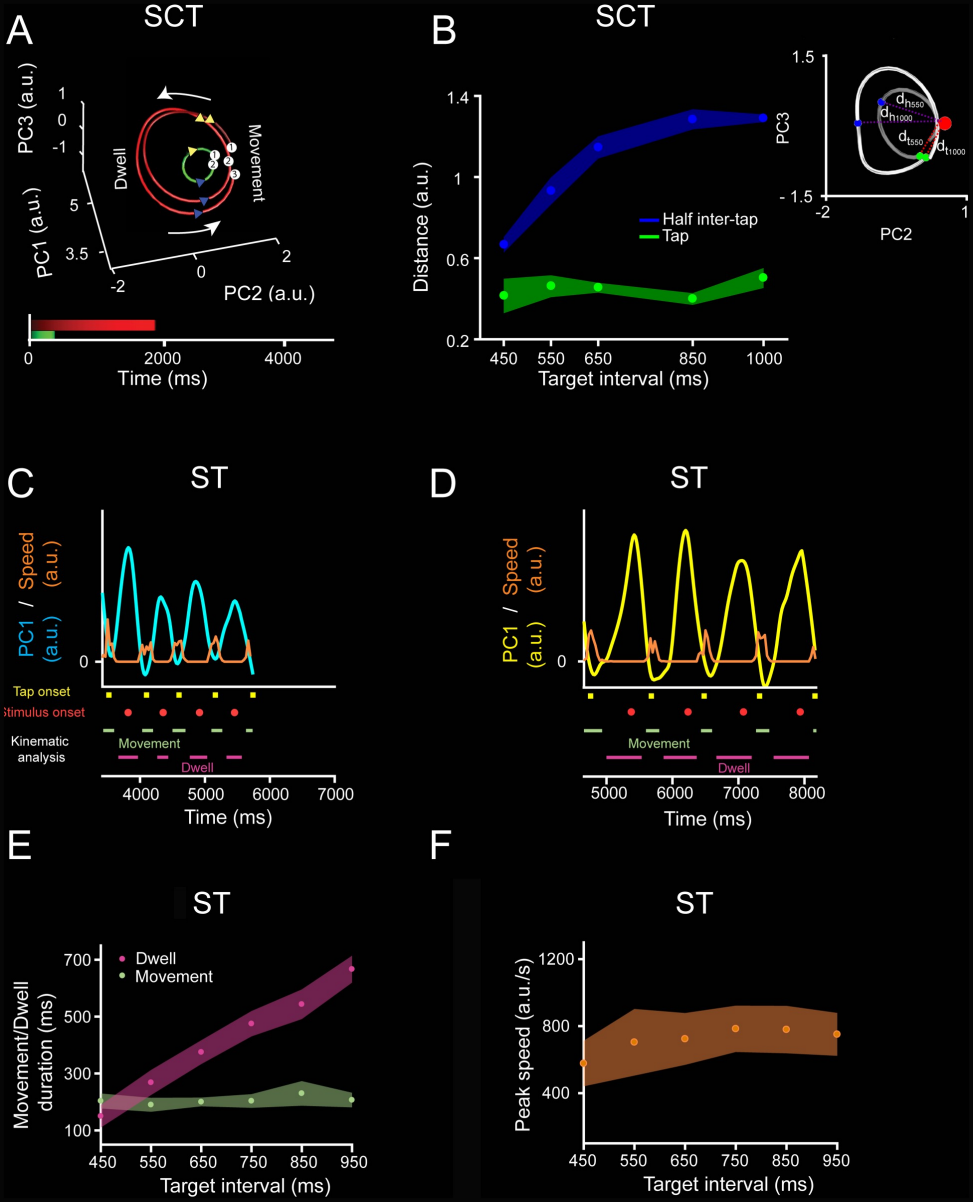
Neural trajectories do not follow the tapping kinematics. **A**. Diagram of the rotational trajectory of the SCT neural activity during three inter-tap intervals: one 450-ms interval (green) and two 1,000-ms intervals (red). Each tap is numbered and projected in the trajectory as a white circle. A blue triangle marks the beginning, whereas a yellow triangle marks the end of the movement time. The monkeys produced phasic stereotypic movements whilst timing the dwell between taps during SCT [37]. **B.** Euclidean distance (d_t_, see inset) between an anchor point (red) and the position of each tap (green, mean ± SD, slope = 0.00007, *R*^2^ = 0.0633, p = 0.225), or half of the inter-tap interval position on the neural trajectories (blue, mean ± SD, slope = −0.001, *R*^2^ = 0.801, *p* < 0.0001) across target intervals for SC. A two-way ANOVA detected significant main effects on position (F(1, 40) = 1855.72, *p* < 0.0001), target interval (F(4, 40) = 77, *p* < 0.0001) and their interaction (F(4, 40) = 63.68, *p* < 0.0001). Tukey HSD post hoc test showed that the distances of the anchor point to tap and half inter-tap positions were significantly different (*p* < 0.05). In contrast, the anchor to tap distances across target intervals were not statistically different. Inset: scheme of the distance calculation; red sphere marks the anchor point and two-sample inter-tap trajectories for 550 ms (dark gray) and 1,000 ms (light gray) are shown. The green sphere marks the tap position and the blue sphere marks the half inter-tap position. Thus, the neural trajectories converge on an attractor around the tap time, to later diverge at half the inter-tap interval. Note that these results suggest the existence of tangent circular trajectories that converge in an intersection zone close to the tapping moment, although their amplitude changed as a function of interval. **C**. Speed of the tapping movement (orange trace) from the second to the sixth tap of ST, and the PC1 projected neural information (cyan) for 26 simultaneously recorded neurons during a trial with a target interval of 550 ms. Taps were represented as yellow squares and stimuli as red circles. Movement and dwell times are depicted in green and magenta, respectively. **D**. Similar to (**C**) during an 850-ms target interval (PC1 projected neural information as a yellow trace). **E.** Mean ± SD of the duration of the movement (green) and the dwell between movements (magenta) across target intervals, computed from the speed profile of the tapping movements. A two-way ANOVA showed significant main effects on kinematic state (movement/dwell duration, F(1, 228) = 1,850.61, *p* < 0.0001), target interval (F(5, 228) = 272.72, *p* < 0.0001), and their interaction (F(5, 228) = 236.18, *p* < 0.0001). Tukey HSD post hoc test showed that dwell durations across intervals were significantly different (*p* < 0.05). Therefore, the monkey modulated the dwell duration to successfully temporalize her behavior, while the down-push-up sequence of the tapping movement was phasic and stereotypic across target intervals. **F.** Mean ± SD of the peak speed during the tapping movement as a function of the target interval during ST (ANOVA main effect interval, F(5, 114) = 5.13, *p* < 0.001). The Tukey HSD post hoc test showed that only the peak speed of the 450-ms target interval trials were significantly different from the 650-, 750-, 850-, and 950-ms trials (*p* < 0.05). Underlying data are available in https://doid.gin.g-node.org/d315b3db0cee15869b3d9ed164f88cfa/. a.u., arbitrary unit; d_h_, Euclidean distance of the anchor point to the half inter-tap position; d_t_, Euclidean distance from the anchor point to the tap position; HSD, honestly significant difference; PC, principal component; SCT, synchronization-continuation task; ST, synchronization task.

The cyclic and smooth nature of the neural trajectories during ST and SCT sharply contrast with the kinematics of movement (Fig 5A, 5C and 5D), which is characterized by stereotypic tapping movements separated by a dwell period that increased as a function of the target interval (Fig 5E; [16,37]). These observations suggest that during rhythmic tapping, an explicit timing mechanism in MPC keeps track of the dwell time by setting in motion a continuous and periodic change in the neural population state. According to this scheme, the tapping command is triggered once the state trajectories get to a specific position in the phase-space that corresponds to the intersection point between the tangent circular paths whose radii increase with the tapping tempo. To test the hypothesis, we computed the distance between a point in state space and the position of the taps in the neural trajectory and found a similar distance across target intervals (Fig 5B, see inset). In addition, the distance between the same point and half inter-tap position increased as a function of target interval (Fig 5B). Therefore, these results support the idea that the neural trajectories behave as tangent circles and encode the dwell time between taps in the PC amplitude and trigger the stereotypic tapping movements once the neural dynamics reach a point in state space (S5 Fig).

### Distributed nature of the trajectories’ timing information

**Fig 6:**
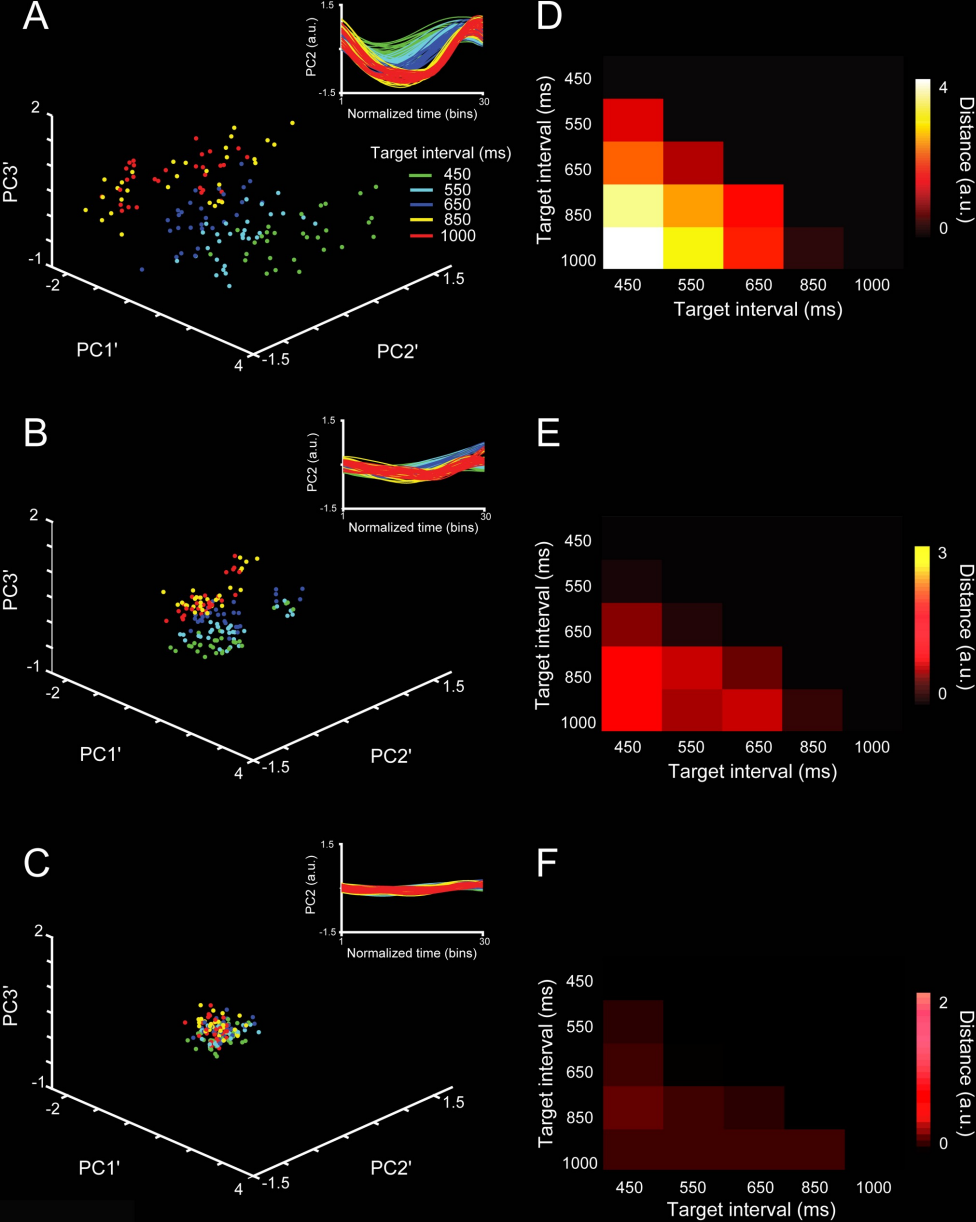
Robustness in the classifier for SCT target interval using segments of the PCA neural trajectory between taps with different neural population sizes. **A-C.** Three principal components projection of the second-layer PCA′ applied to each of the six inter-tap neural trajectory segments and the five trial repetitions (see inset) for (**A**) 100%, (**B**) 50%, and (**C**) 1% of the neural population. Each dot in the second-layer PCA′ corresponds to an inter-tap trajectory segment. Target interval color in the inset in (**A**). **D-F.** Distances between cluster centroids of data projection across target intervals for (**D**) 100%, (**E**) 50%, and (**F**) 1% of the neural population. Underlying data are available in https://doid.gin.g-node.org/d315b3db0cee15869b3d9ed164f88cfa/. a.u., arbitrary unit; PC, principal component; PCA, principal component analysis; SCT, synchronization-continuation task.

**Fig 7:**
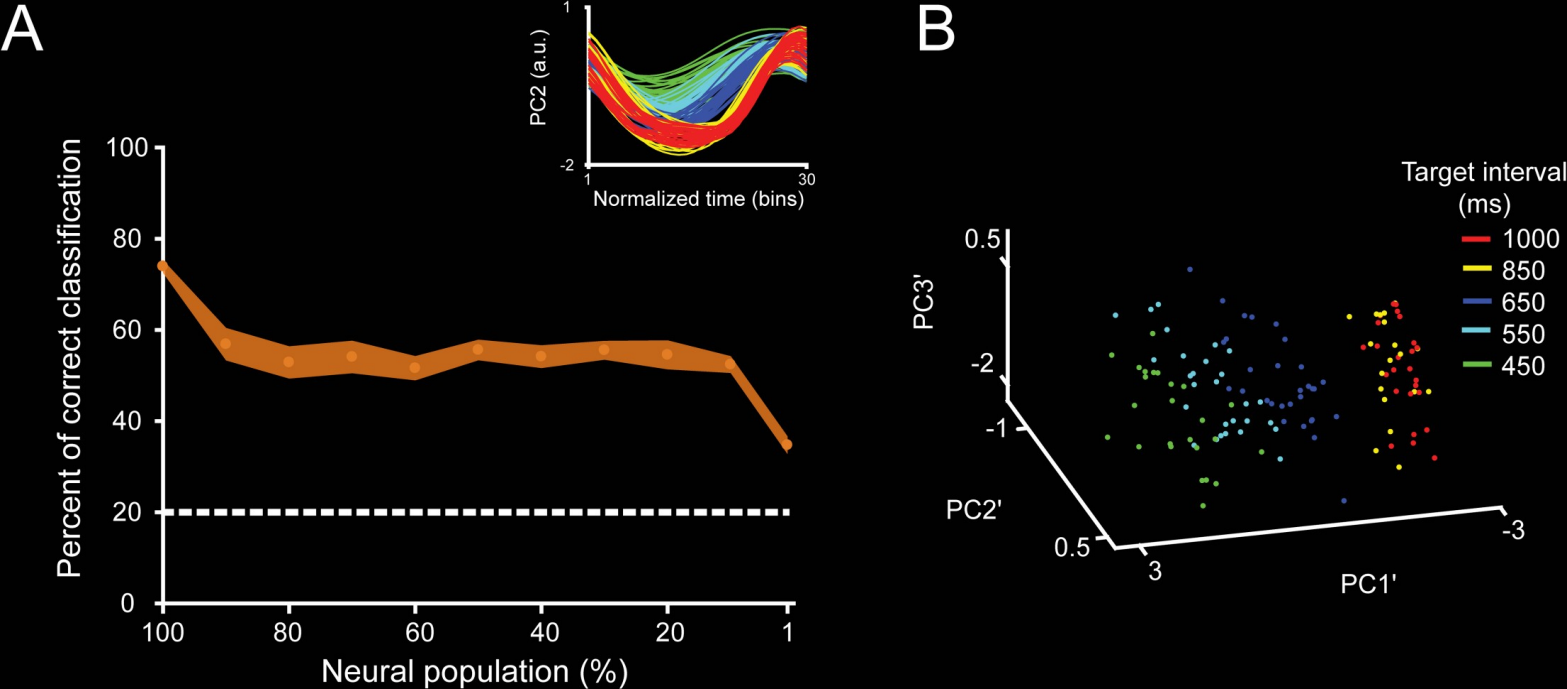
Trajectory classifier robustness across neural population sizes during SCT. **A**. SVM classifier performance (mean ± SD of percent of correct classifications) for target interval (five instructed intervals) during the SCT task based on the neural trajectory computed from different population sizes. The total initial population size was of 1,477 neurons. Dotted lines correspond to random level. The neurons with the largest PC participation were removed in steps of 10% of the original population size, until reaching 1% of the original population. Inset shows the original time-normalized neural trajectory PC used to generate the second-layer PCA′. **B**. Point cloud in 3D for the second-layer PCAs’ for target interval. See color code in the inset. Note that the percentage of correct classification decreased as a function of the population size; however, the classification was above chance even for the trajectories based on small cell ensembles. Underlying data are available in https://doid.gin.g-node.org/d315b3db0cee15869b3d9ed164f88cfa/. a.u., arbitrary unit; PC, principal components; PCA, principal component analysis; SCT, synchronization-continuation task; SVM, support vector machine.

We determined whether we could extract information about the target interval from the neural population dynamics, and how this information was modulated by the size of the neural population used to compute the trajectories. To this end, we first segregated each segment of the single-dimension trajectory according to the SCT target interval (450, 550, … 1,000 ms; see insets in Fig 6A). Then, to capture the shape of the trajectory segments as a single three-dimensional coordinate, we applied a second-layer PCA (PCA′) and kept the first three PCs. As a result, we obtained a dot cloud in 3D, in which each point represents a particular produced interval trajectory segment (Fig 6A). We trained support vector machines (SVMs) to classify the cloud of points for the five target intervals of the SCT. We trained the SVM ten times and used 5-fold cross-validation to evaluate the performance of the classifier. On the other hand, each neuron was sorted according to the weight magnitude of the original PCAs. The neurons with the largest PC participation were removed in steps of 10% from the original population size, and the second-layer PCAs were computed on the new trajectories. Finally, the SVM was carried out on the second-layer PCAs for different population sizes (see Fig 6). There was an asymptotic decline in the classifier performance with the removal of a larger percentage of the neural population (Fig 7A). However, even with very small populations (total cells: 15), the classifier was able to extract all SCT target intervals above chance. These results are in line with the idea that the temporal structure of rhythmic behavior depends on a neural population code that is distributed within MPC.

### Neural population trajectories and evolving activation patterns

The results of the previous section revealed a distributed representation of tapping tempo across MPC cell populations. However, a critical question is what aspects of the time-varying activity defined the changes in amplitude in the neural trajectories as a function of the timed duration [28]. Based on our previous observations [4,21], we hypothesized that the evolving patterns of neural activity could be directly linked with the time-encoding features of the neural trajectories during the SCT. Consequently, to test this idea we first characterized the properties of neuronal moving bumps [21,23,36] during this task. With this information we carried out simulations to determine whether the key features of the moving bumps were linked to the observed changes in curvature radius and variability as a function of duration in the neural state trajectories.

**Fig 8:**
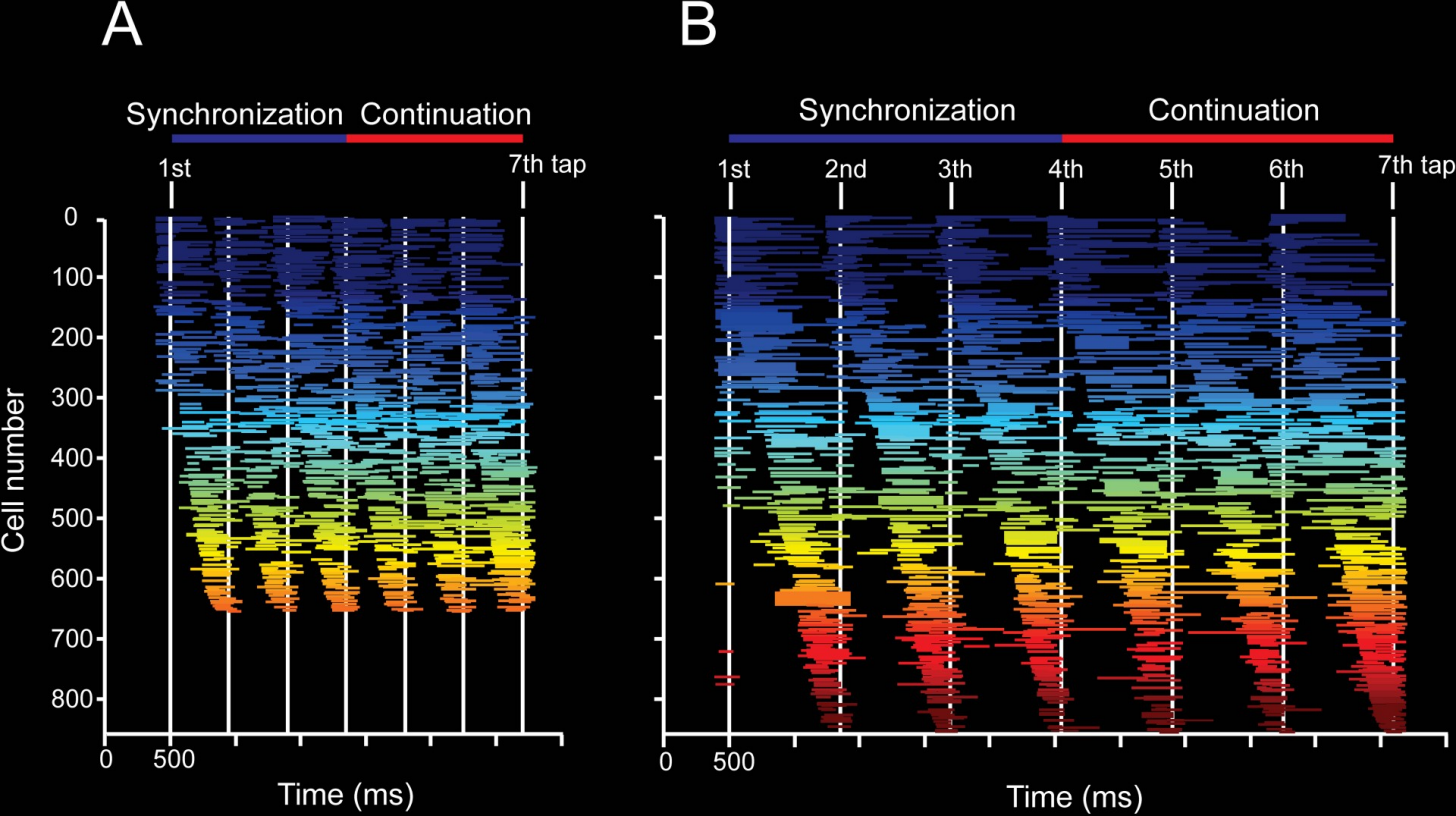
Overall patterns of activity in cell populations. **A,B.** Neural activation periods, sorted by their mean peak activation time, during the SCT task for the target intervals of 450 (A) and 850 (B) ms. Each horizontal line corresponds to the onset and duration of the significant activation period of a cell according to the Poisson-train analysis (see Materials and methods). The Poisson-train analysis was carried out on the discharge rate of cells that was warped in relation to the tapping times (seven white vertical lines [4,73]). Note that the number of cells with significant activation periods is larger for the longer target interval. Underlying data are available in https://doid.gin.g-node.org/d315b3db0cee15869b3d9ed164f88cfa/. SCT, synchronization-continuation task.

**Fig 9:**
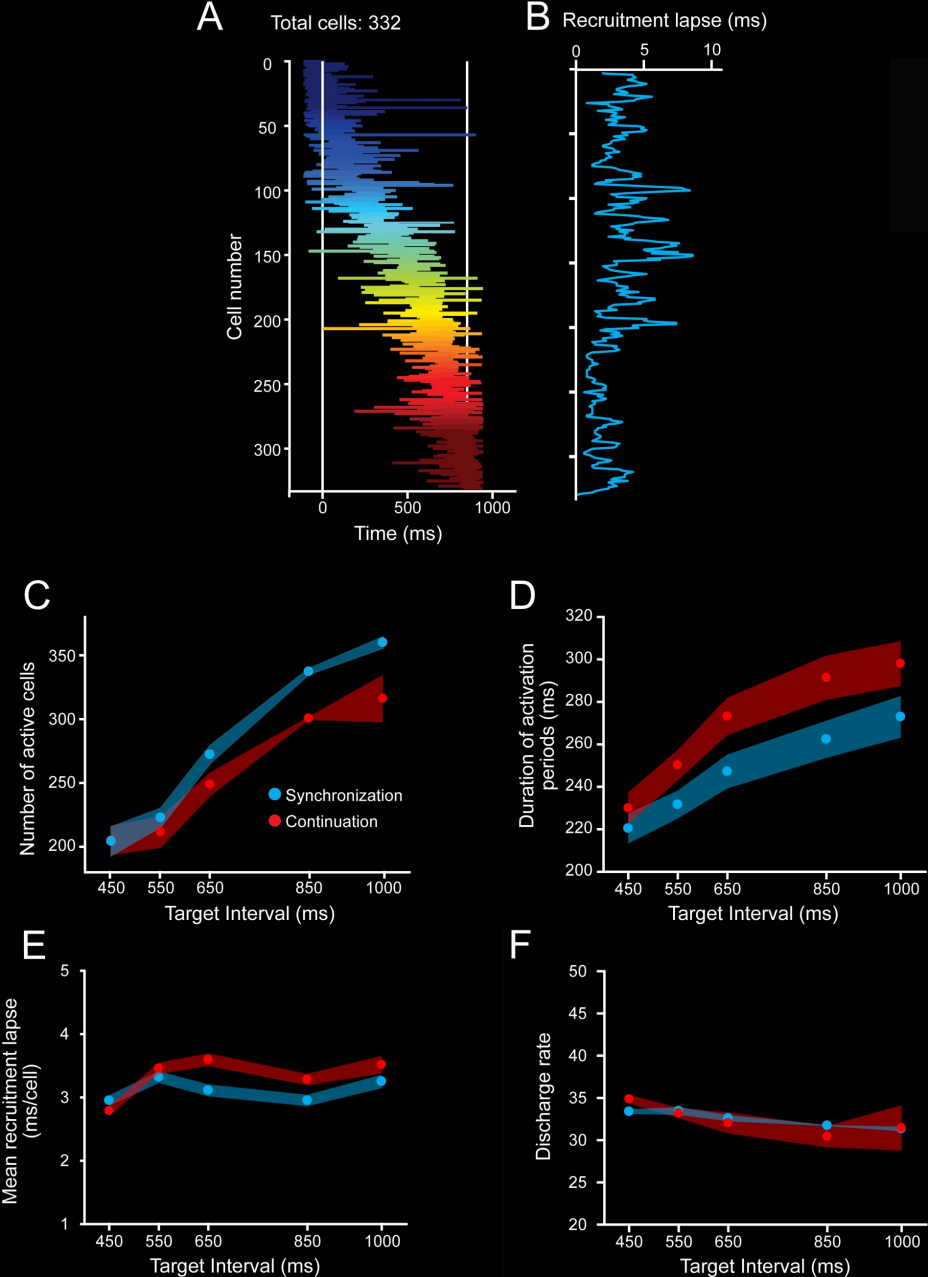
Evolving patterns of activation. **A.** Neural activation periods for the second produced interval (second and third taps as white vertical lines) during SC for the target interval of 850 ms. The horizontal lines of each row correspond to the onset and extent of the activation periods detected by the Poisson-train analysis. Cells were sorted by their time of peak activity. **B.** Recruitment lapse as a function of cell number. The activation lapse was the difference in the time of peak activity between contiguous cells in the neural avalanche. The mean activation lapse (±SEM) was 2.98 ± 0.08 ms. **C.** Number of cells with significant activation periods across target intervals for SC (blue) and CC (red). Avalanches for longer intervals recruited more cells (ANOVA main effect target interval, F(4, 20) = 21.1, *p* < 0.0001; main effect task condition, F(1, 20) = 6.2, *p* < 0.02; interval × condition interaction, F(4, 20) = 0.71, *p* = 0.594). **D.** Duration of the activation periods during the SC (blue) and CC (red) increased as a function of target intervals. (ANOVA main effect target interval, F(4, 20) = 18.9, *p* < 0.0001; main effect task condition, F(1, 20) = 26.7, *p* < 0.0001; interval × condition interaction, F(4, 20) = 1.3, *p* = 0.268). **E.** Mean neural recruitment lapse during SC (blue) and CC (red) did not change as a function of target interval (ANOVA main effect target interval, F(4, 20) = 2.7, *p* = 0.06; main effect task condition, F(1, 20) = 3.4, *p* = 0.08; interval × condition interaction, F(4, 20) = 0.79, *p* = 0.55). **F.** The discharge rate during activation periods in SC (blue) and CC (red) did not vary across target intervals (ANOVA main effect target interval, F(4, 20) = 2.2, *p* = 0.06; main effect task condition, F(1, 20) = 0.86, *p* = 0.35; interval × condition interaction, F(4, 20) = 0.92, *p* = 0.45). Underlying data are available in https://doid.gin.g-node.org/d315b3db0cee15869b3d9ed164f88cfa/. CC, continuation condition; SC, synchronization condition.

As expected, a substantial proportion of MPC cells during the SCT showed a progressive pattern of activation in the neuronal population, consisting of a gradual response onset of single cells within a produced interval (Fig 8, see Materials and methods). This activation pattern started before a tap, migrated during the timed interval, and finished after the next tap (Fig 8). In addition, a similar response profile was repeated in a cyclical manner for the three intervals of SC and the three intervals of CC (Fig 8A and 8B) [4,21]. These findings suggest that rhythmic timing can be encoded in the sequential activation of neural populations [23]. A central question is what parameters of the neuronal response profiles are encoding the target interval and the SCT condition. Remarkably, the number of neurons involved in these evolving activation patterns (Fig 8A and 8B, Fig 9C), as well as the duration of neural activation periods (Fig 9D), increased as a function of the target interval. SC showed a larger number of active cells, whereas CC showed a longer activation period. In contrast, the neural recruitment lapse, namely the time between pairs of consecutively activated cells (Fig 9E), and the cells’ discharge rate (Fig 9F) did not show statistically significant changes across target intervals and task phases. These results suggest that both the size of the circuits involved in measuring the passage of time and the duration of their activation times are core time-encoding signals in MPC, and suggest the existence of a delicate balance between these two measures to produce the progressive activation profiles of neurons when tapping to a metronome or an internally generated rhythmic signal (Fig 9C and 9D).

**Fig 10:**
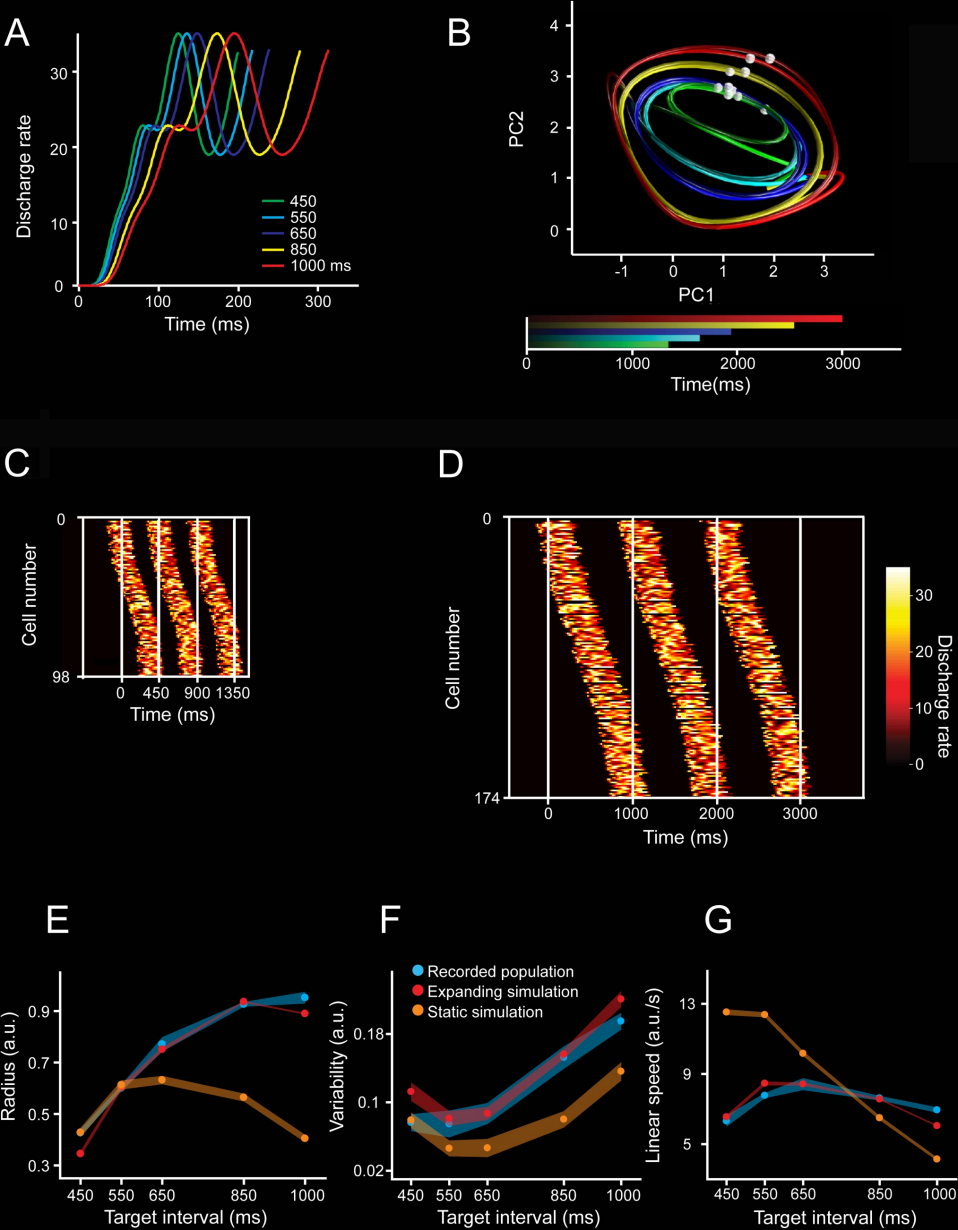
Simulations of moving bumps and neural trajectories. **A.** Activity profile of one simulated neuron during its activation period is scaled for the five simulated durations. **B**. Neural trajectories generated from the population activity of moving bumps simulations. The number of neurons and activation periods varied across intervals (see Materials and methods). The simulated interval is color coded. Second and third simulated taps are marked as white spheres on each trajectory. **C,D**. Activation profiles of neurons for three consecutive simulated intervals with durations of 450 ms (C) and 1,000 ms (D). The white vertical lines correspond to the tap events defining the intervals. The activation profiles follow a Gaussian shape of cell recruitment, with slow activation rates at the tails (close to each tap). The number of neurons and the duration of the activation periods increased as a function of simulated interval. **E,F,G.** Radii (E), variability (F), and linear speed (G) of the neural trajectories generated from simulations. Data from the simulated neural activity with growing numbers of neurons and activation periods (expanding simulation: red), constant duration of activation periods and constant number of neurons (static simulation: orange), and from the actual recorded population during SCT (blue) across target intervals. Note that a constant was added to both simulation data in graphs. (E) Radii for simulation with expanding parameters (red, mean ± SD, slope = 0.0009, *R*^2^ = 0.811, *p* < 0.0001), simulation with static parameters (orange, mean ± SD, nonsignificant linear regression, slope = −0.0001, *R*^2^ = 0.811, *p* = 0.214), and actual neural activity (blue, mean ± SD, slope = 0.0009, *R*^2^ = 0.897, *p* < 0.0001). The slopes of the radius, variability, and linear speed were not statistically different between the simulations with expanding parameters and the actual neuronal trajectories (radius slope *t* test = 0.15, *p* = 0.878; variability slope *t* test = 0.25, *p* = 0.803; linear speed slope *t* test = 1.8, *p* = 0.077). However, the slopes between the simulations with constant parameters and neuronal trajectories showed statistically significant differences (radius slope *t* test = 9.13, *p* < 0.0001; variability slope *t* test = 3.73, *p* < 0.001; linear speed slope *t* test = 17.71, *p* < 0.0001). Underlying data are available in https://doid.gin.g-node.org/d315b3db0cee15869b3d9ed164f88cfa/. a.u., arbitrary unit; PC, principal component; SCT, synchronization-continuation task.

**Fig 11:**
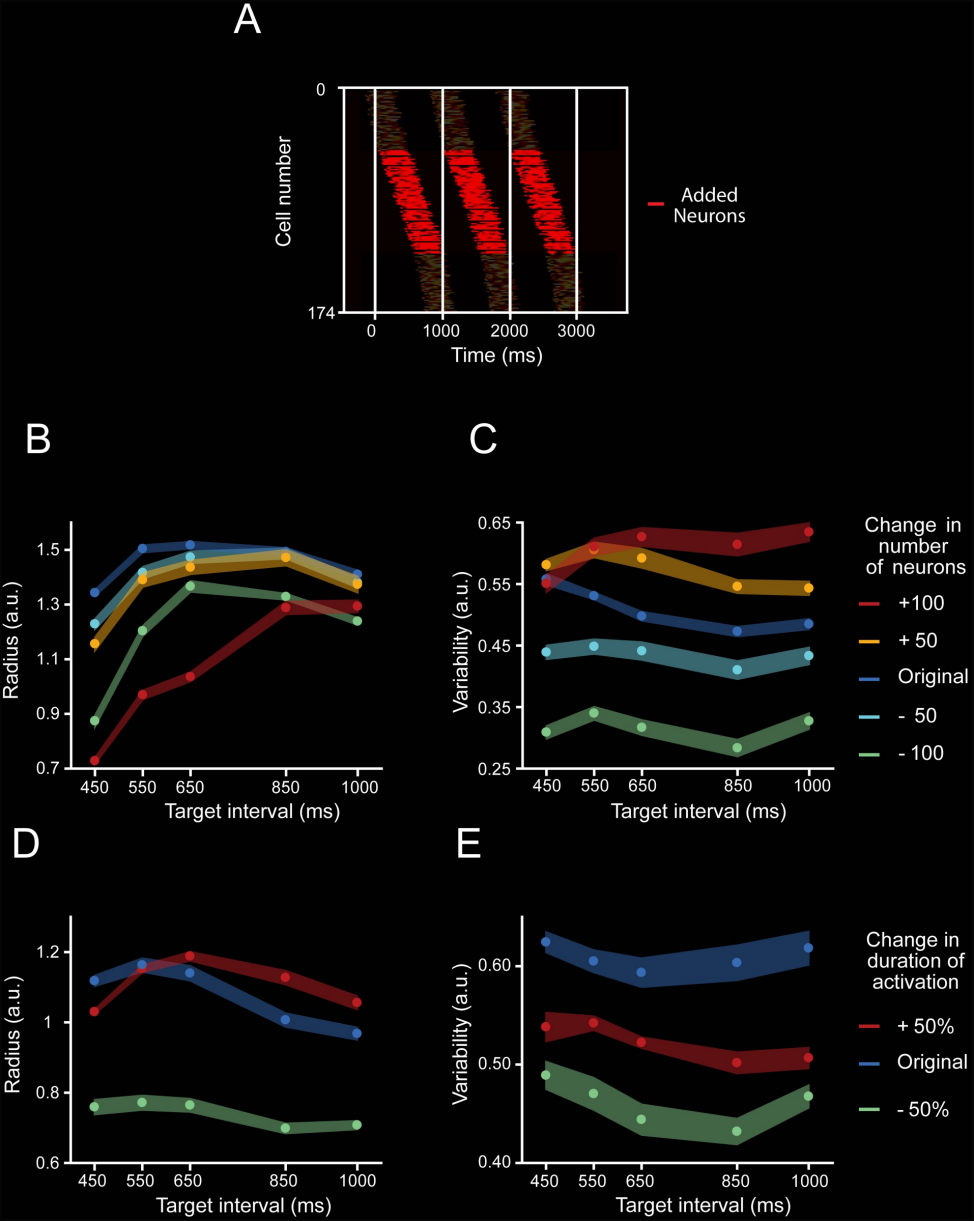
Moving bump simulation parameters. **A.** Temporal positions of the activation periods of the neurons that were included to the simulation of the 1,000-ms target interval trial (red), in addition to the position of the activation periods of neurons that also participated in the 450-ms simulation (shaded). **B,C.** Radius (B) and variability (C) of PCA trajectories generated from moving bump simulations when the number of neurons was modified by a constant number (−100, green; −50, cyan; +50, yellow; and +100, red) from the original number of neurons (208 neurons for 450-ms target interval, 220 neurons for 550-ms target interval, 230 neurons for 650-ms target interval, 270 neurons for 850-ms target interval, and 282 neurons for 1,000-ms target interval; blue) while the activation period was kept constant at 257 ms across target intervals. A two-way ANOVA on the radius showed significant main effects for number of neurons (F(4, 100) = 10,544.2, *p* < 0.0001), target interval (F(4, 100) = 4,013.12, *p* < 0.0001), and their interaction (F(16, 100) = 25.8, *p* < 0.0001). Tukey HSD post hoc test showed significant differences for the radii of all simulations with different numbers of neurons and for all target intervals (*p* < 0.05). Additionally, A two-way ANOVA on the variability showed significant main effects for number of neurons (F(4, 100) = 2,421.8, *p* < 0.0001), target interval (F(4, 100) = 3,476.91, *p* < 0.0001), and their interaction (F(16, 100) = 22.53, *p* < 0.0001). Tukey HSD post hoc test showed significant differences for the variability of all simulations with different numbers of neurons (*p* < 0.05). **D,E.** Radius (D) and variability (E) of the trajectories generated from neural moving bumps in which the duration of the activation periods was reduced by 50% (short, green) or increased by 50% (long, red) of the original scaled duration (197 ms for 450-ms target interval, 205 ms for 550-ms target interval, 213 ms for 650-ms target interval, 233 ms for 850-ms target interval, and 257 ms for 1,000-ms target interval; blue) while the number of neurons was kept constant at 130 across target intervals. A two-way ANOVA on the variability showed significant main effects for activation duration (F(2, 60) = 3,081.54, *p* < 0.0001), target interval (F(4, 60) = 2,801.16, *p* < 0.0001), and their interaction (F(8, 60) = 211.34, *p* < 0.0001). Tukey HSD post hoc test showed significant differences for all simulations with different activation durations (*p* < 0.05). In addition, a two-way ANOVA on the variability showed significant main effects for activation duration (F(2, 60) = 1,227.53, *p* < 0.0001), target interval (F(4, 60) = 257.49, *p* < 0.0001), and their interaction (F(8, 60) = 24.87, *p* < 0.0001). Tukey HSD post hoc test showed significant differences for all simulations with different activation durations (*p* < 0.05). Thus, the number of neurons and the activation duration within moving bumps produce large changes in the radius and variability of the simulated neural trajectories. a.u., arbitrary unit; HSD, honestly significant difference; PCA, principal component analysis.

Next, we simulated evolving patterns of population activity with different response profiles and evaluated their translation onto PCA state space. First, we generated activity patterns on individual units that were complex, heterogenous, and that scaled in time, producing activation periods with the same time-varying activity but different durations (Fig 10A, see Materials and methods) [24]. Then, we simulated population cascade patterns for three consecutive intervals, emulating two key features on the MPC population responses: a gradual response onset of single cells that started before, migrated within, and finished after the end of an interval, with a constant overall recruitment of cells over time; and the cyclical repetition of this response profile for the three intervals (Fig 10C and 10D). In addition, Fig 11A shows that neurons were added randomly in the intermediate portion of the simulated moving bumps when increasing the total number of neurons. The projection of the simulated cascades onto PCA space produced oscillatory trajectories (Fig 10B), whose radii and variability increased but the linear speed was similar with the target interval, as seen in the actual population responses. Importantly, these properties were only followed when the simulated neural cascades included an increase in both the number of neurons and the duration of the activation periods as a function of target interval (Fig 10E and 10F). Simulations with constant values in both parameters produced PCA trajectories with similar radii or variability across interval durations, and a decrease in speed with target interval consistent with the notion of temporal scaling (Fig 10E–10G, Fig 11B–11E). Furthermore, the scaling of the response duration alone did not reproduce the observed changes in radii and variability across durations in the state trajectories (Fig 11D–11E). These findings indicate not only a close relation between the properties of the sequential neural patterns of activation and the neural state trajectories during rhythmic tapping, but also suggest that an increment in the number of neurons engaged in the evolving patterns of population activity is fundamental to reproducing the two critical duration-dependent features of the PCA neural population trajectories: the increase in the magnitude and variability of the radii as a function of target interval.

## Discussion

The present study supports four conclusions. First, the time-varying discharge rate of MPC cells shows a strong periodic organization when projected onto a two-dimensional state space, generating a circular neural trajectory during each produced interval. The amplitude of this trajectory increases with target duration and is closely related to the rhythmic tapping during the SCT and ST, but not during the reactive tapping of SRTT. Second, the scalar property, a hallmark of timing behavior, was accounted for by the variability of the curvilinear radii in the PCA neural trajectories. Third, the population dynamics for simultaneously recorded MPC cell populations during ST contained information to accurately decode the tapping times on a trial-by-trial basis. Last, there is a strong correlation between the interval-associated changes in radial magnitude and variability of the periodic neural trajectories during SCT and the number of neurons involved in the sequential activation patterns, as well as the duration of their transient periods of activation within these moving bumps.

### Rhythmic timing and the amplitude of neural state trajectories

The network state trajectories showed the following properties: they were simple, periodic, exhibited an amplitude modulation according to the timed duration, and were different from the stereotypic kinematics of the phasic tapping movements and the timing control of the dwell between movements in this task [16,37]. Notably, the increases in trajectory amplitude as a function of target interval were observed during the two rhythmic tapping tasks, reproduced with dPCA, and closely related with the monkeys’ produced intervals during SCT and ST. Furthermore, the switch from predictive rhythmic tapping to a reaction time task (SRTT) produced a profound disorganization in the periodicity of neural trajectories, accompanied by no changes in radial amplitude. In contrast with the temporal scaling model [24], we found that the neural trajectories do not scale in time, because they present a time-related amplitude modulation with similar linear speed profiles across durations. In line with our observations, neural-network simulations of complex sensorimotor patterns showed that temporal scaling of input stimuli produced curvilinear trajectories that increased in radii for longer intervals [38]. Hence, amplitude modulations in neural population trajectories can be associated with rhythmic timing [39] or complex temporal processing [38].

We found a strong correlation between the duration of the produced intervals and the curvilinear amplitude of the MPC neural trajectories during the SCT and ST, and, due to the simultaneity of the recordings in the latter task, we decoded accurately the produced durations on a trial-by-trial basis. In addition, the cyclic and smooth nature of the neural trajectories during ST and SCT sharply contrasts with the tapping kinematics, which are characterized by stereotypic tapping movements separated by a dwell period that increases with the timed interval [16,37]. Previous studies have demonstrated that cell populations in premotor and motor cortical areas show rotatory non-muscle-like trajectories that reflect the internal dynamics needed for controlling reaching and cycling [40,41]. Under this scenario, we found evidence supporting the notion that the periodic MPC trajectories during rhythmic tapping encode the dwell between taps in their curvilinear radii and that the tapping command is triggered whenever the trajectory reaches a specific phase-space, which corresponds to the intersection point between the tangent circular paths. This dynamical geometry contrasts with the neural trajectories of medial frontal areas during a single interval reproduction task [34]. In this interval-based paradigm, the state trajectories not only evolve at different speeds but also generate parallel paths for different timed intervals, depending on the initial conditions of the neural population dynamics [34]. Thus, the present data are consistent with the notion that timing is encoded in a neural population clock [28,42–45] and puts forward the hypothesis that temporal processing during the entrainment to an isochronous metronome depends on the amplitude of tangent circular trajectories in MPC populations. Under this scenario, temporal processing is governed by MPC neural population clocks that switch from temporal scaling of their state dynamics during interval timing to amplitude modulation in their tangent circular trajectories during rhythmic timing. Importantly, because MPC is part of both the cortico-basal ganglia and the cortico-cerebellar circuits, it can play an important role in both interval and rhythmic timing and can act as a synergistic context-dependent element within the two core timing systems, as suggested previously [46–49].

Beat perception in humans is shaped by the temporal structure of extrinsic musical sound and by the metrical interpretation that defines where a subject hears the beat. Thus, the perception of a beat and the corresponding movement entrainment depend on a mental interpretation of the metrics of music. The Dynamic Attending Theory (DAT) is one of the most successful hypotheses to explain these phenomena. According to DAT, it is possible to match the tapping movements to a beat during rhythmic entrainment because the periodic dynamics of music drive our attention [50,51], allowing the prediction of the next pulse in the rhythmic auditory sequence. The DAT suggests that attention is a dynamic process that can be successfully modeled by internal self-sustained oscillations in the auditory system [52,53]. These internal oscillations generate periodic shifts in attention to the most salient events in the sound signal (the pulse that constructs an isochronous sequence in the musical stream), so that the brain generates rhythmic expectations that correspond to the subjective interpretation of the beat. Indeed, electroencephalogram recordings in auditory areas of humans have shown that the brain oscillates at both the exogenous frequency of stimuli and at the metric interpretation of the beat, providing strong support for DAT [54]. In addition, the perception of an inferred musical beat in humans strongly engages the motor system, including the basal ganglia and the MPC [55,56], supporting the notion that rhythmic perception and entrainment depend on a dynamic interaction between the auditory and motor systems in the brain [15,43,57]. Consequently, the present findings add important elements to these ideas, namely, neural populations in the motor system show cyclic dynamics whose period is tightly associated with the tempo of the isochronous metronome, even when the metronome is turned off and the monkeys continue tapping with the same tempo. Hence, in accordance with DAT, the MPC neural trajectories act as a neural oscillator, with a period similar to the tapping tempo during both the sensory cued and the internally driven rhythmic tapping. Furthermore, in agreement with the audiomotor hypothesis for beat perception and entrainment, our data suggest that preSMA and SMA generate a periodic and predictive neural population signal that not only times the inter-tap dwell and triggers the rhythmic tapping movement, but also may help the sensory system to expect a specific temporal structure on the metronome [57,58]. However, a couple of cautionary notes are in place here. First, monkeys can perceive and predictively synchronize to isochronous metronomes [11,16,59]. We still do not know what the metrical hierarchy is that monkeys can perceive and entrain to [11], but, definitively, nonhuman primates do not have the flexibility to predictively perceive and entrain to a pulse across the range of tempi and meters observed in humans [6]. Hence, our present data may generalize only to isochronous rhythmic timing in humans. Second, monkeys show a bias to synchronize to visual rather than auditory metronomes [16], whereas humans have a strong entrainment bias towards auditory sequences, including music [1,14]. It has been suggested that the connections between the dorsal auditory regions and the motor planning areas via parietal cortex are stronger in humans than in nonhuman primates, conferring the latter their larger ability for beat perception and entrainment [15,57]. Therefore, it is quite possible that the neural state dynamics in the audiomotor system of the *Homo sapiens* are more flexible and complex than what we report here.

### The scalar property of timing and the state dynamics variability

The scalar property states that temporal variability increases linearly as a function of timed duration [60]. This hallmark feature of temporal processing has been documented across many timing tasks and species [20,60–63]. Several computational models based on neural population time representations have been implemented to describe this property, including drift diffusion [64,65] and recurrent networks [36,66]. Here, we found that the variability in the radii of neural trajectories increased as a function of target interval during SCT and ST, but remained similar during the SRTT, a task that precludes time prediction while preserving the sensory and tapping components. Therefore, these results suggest that the amplitude of the MPC state-network trajectories is a feasible neural correlate of the scalar property during rhythmic tapping.

### The relation between neural trajectories and moving bumps during rhythmic tapping

The dynamics of coordinated neural population activity define the evolution of the network state trajectories, which in turn have revealed functional principles in a variety of behaviors that are not evident at the single cell level [24,30,32,67]. Notably, the tapping tempo is strongly mapped in the neural trajectories and is encoded in a distributed fashion, not dependent on a particular response profile of individual neurons. Within this neural population framework, we found large groups of neurons that showed sequential transient activation patterns that traversed each produced interval during the SCT. Previous studies have reported moving bumps as a timing mechanism in parietal cortex [68], MPC [4,21], the basal ganglia [23,69,70], and hippocampus [71,72]. For example, the bump activity in the rat striatum during a peak interval task moved progressively slower as the timed interval progressed, providing a functional basis for the decrease in the animals’ timing accuracy as the length of the timed interval increased [23]. In contrast, during the SCT we found that the rate of engagement of the neurons within moving bumps was constant and was accompanied by an increase in the number of neurons participating in the evolving patterns of population activity. Thus, an optimal reader could estimate the tempo of rhythmic tapping based on two signals: the location of the activity within a bump, in which longer intervals engaged moving bumps composed of a larger number of neurons, and the resetting between consecutive evolving activation patterns [65]. Strikingly, our simulations revealed a tight relation between the scaling of the duration of the transient period of activity, the increase in the number of neurons within moving bumps, and the increase in radius and variability of the corresponding neural trajectories. The simulations also suggest that neurons have the same relative position within a moving bump independently of the timed interval, as seen previously in the rat striatum [23]. Consequently, the increase in neural population size for longer intervals implies incorporation of new cells at intermediate locations within the moving bump [21]. These results not only replicate our empirical observations but also support the notion that the properties of moving bumps, especially the number of participating neurons, can shape the curvilinear amplitude and the corresponding variability in neural state trajectories during SCT.

## Conclusions

Overall, these findings support the notion that the rhythmic timing mechanism is based on the changes in curvature radii of the neural population state dynamics in MPC, with slower tempos encoded in larger traversed distances in the tangent periodic neural trajectories, and suggest that the variability in these neural trajectories is a feasible neural substrate of the scalar property during rhythmic tapping.

## Materials and methods

### Ethics statement

All the animal care, housing, and experimental procedures (protocol 090.A INB) were approved by the Ethics in Research Committee of the Universidad Nacional Autónoma de México and conformed to the principles outlined in the Guide for Care and Use of Laboratory Animals (NIH, publication number 85–23, revised 1985).

### Subjects

Two monkeys (M01 and M02, *Macaca mulatta*, both males, 5–7 kg BW) were trained to tap on a push button in SCT, ST, and SRTT. The monkeys were monitored daily by the researchers and the animal care staff to check their conditions of health and welfare.

### Tasks

#### SCT

The SCT has been described before [14]. Briefly, the monkeys were trained to push a button each time stimuli with a constant interstimulus interval were presented. This resulted in a stimulus-movement cycle (Fig 1A). After four consecutive synchronized movements, the stimuli were eliminated, and the monkeys had to continue tapping with the same interval for three additional intervals. Monkeys received a reward (drops of juice) if each of the intervals produced had an error <30% of the target interval. The daily performance of the monkeys was >70% of correct trials. The amount of juice was proportional to the trial length. Trials were separated by a variable intertrial interval (1.2–4 s). The target intervals, defined by visual stimuli (red square with a side length of 5 cm, presented for 33 ms), were 450, 550, 650, 850, and 1,000 ms. The target intervals were chosen pseudorandomly within a repetition. Five repetitions were collected for each target interval.

#### ST

This task was similar to the synchronization phase of the SCT [16]. The subject had to push a button with a stimulus. Six stimuli with a constant interstimulus were presented (red square with a side length of 5 cm, shown for 33 ms). Thus, the metronome was always present during the task. The target intervals were 450, 550, 650, 750, 850, and 950 ms. Five repetitions were collected for each target interval.

#### SRTT

This task is also described elsewhere [14]. Monkeys were required to push a button each time a stimulus was presented, but in this case the interstimulus interval within a trial was random (picking randomly from the same 450, 550, 650, 750, 850, or 950 ms), precluding the explicit temporalization of tapping (Fig 1B). Monkeys received a reward if the response time to each of the five stimuli was within a window of 200 to 500 ms. The intertrial interval was as ST. Visual (white square with a side length of 5 cm, presented for 33 ms) stimuli were used, and five repetitions were collected.

### Neural recordings

For the SCT, the extracellular recordings were obtained from the MPC of the monkeys using a system with 7 or 16 independently movable microelectrodes (1–3 MΩ, Uwe Thomas Recording, Germany, S3). Only correct trials were analyzed. All isolated neurons were recorded regardless of their activity during the task, and the recording sites changed from session to session. At each site, raw extracellular membrane potentials were sampled at 40 kHz. Single-unit activity was extracted from these records using the Plexon offline sorter (Plexon, Dallas, TX). Using the seven-electrode system, the number of simultaneously recorded cells ranged from 5 to 14 cells, whereas with the 16-electrode system the number ranged from 10 to 35 cells during a recording session. In the present paper we analyzed the activity of 1,477 (1,074 of Monkey 1 and 403 of Monkey 2) MPC neurons in both monkeys. The functional properties of some of these cells (1,083 neurons) have been reported previously [20,21,25]. In addition, using a semichronic, high-density electrode system [35], 26 and 41 MPC cells were recorded simultaneously while Monkey 1 was performing the ST and SRTT tasks. All the isolated neurons were recorded regardless of their activity during the SCT, ST, and SRTT, and the recording sites changed from session to session.

### Neural activation periods

We used the Poisson-train analysis to identify the cell activation periods within each interval defined by two subsequent taps. This analysis determines how improbable it is that the number of action potentials within a specific condition (i.e., target interval and ordinal sequence) was a chance occurrence. For this purpose, the actual number of spikes within a time window was compared with the number of spikes predicted by the Poisson distribution derived from the mean discharge rate during the entire recording of the cell. The measure of improbability was the surprise index (*SI*), defined as follows:

SI=−logP

where *P* was defined by the Poisson equation:

$$P=e^{-rT}\sum_{i=n}^{\infty }\frac{{\left(rT\right)}^{i}}{i!}$$

where *P* is the probability that, given the average discharge rate *r*, the spike train for a produced interval *T* contains *n* or more spikes in a trial. Thus, a large *SI* indicates a low probability that a specific elevation in activity was a chance occurrence. This analysis assumes that an activation period is statistically different from the average discharge rate *r*, considering that the firing of the cell is following a nonhomogenous Poisson process (see also [73]). The detection of activation periods above randomness has been described previously [4,74]. Importantly, the Poisson-train analysis provided the response-onset latency and the activation period for each cell and for each combination of target interval/serial order.

### Neural trajectories

#### Event time normalization and binarization

We developed a time-normalization algorithm to align the neural data from different tapping times of different recording sessions in the same relative time framework. For each neuron, we calculated the produced interval (time between two taps). Then, we subtracted the time of the second tap of a produced interval in the task sequence from all spike and stimulus times (event_times_) and divided them by the produced interval. The tapping times acquired values of minus one and zero, and all the other event_times_ were normalized between these two values. Finally, we added the tap sequence number. Thus, all the normalized values for movement, sensory, and spike events acquired values between zero and seven in an SCT trial, as follows:

$$time\_normalized\_event=\frac{\left(event\_time-tap\_time\right)}{produced\_interval}+tap\_sequence$$

Therefore, the time range of events between the first and the last tap of the normalized data of a trial (unit time normalized data [UTND]) was the same regardless of the target interval. In addition to the trial relative time framework, we also used the target interval normalized data (TIND), which corresponds to the UTND multiplied by the target interval. This time-normalization procedure was not necessary for simultaneously recorded data.

#### Trial binarization

For UTND, TIND, and simultaneously recorded data, we divided the neural data in bins by calculating the discharge rate on consecutive windows of 0.02 units. For UTND, we always got 50 bins between each pair of taps across target intervals, whereas for TIND and the simultaneously recorded data, this number depended on the target interval of the trial. For example, the total number of bins was 23 and 50 for trials with the 450- and 1,000-ms intervals, respectively. The binned data of each neuron were divided by the maximum discharge rate of that particular neuron across all repetitions and target intervals of the SCT. We did not use this time-normalization algorithm on the ST and SRTT data.

#### Principal component coefficients matrix

Given a linear transformation of a matrix **X** into a matrix **Y**, such that each dimension of **Y** explains variance of the original data **X** in descending order, PCA can be described as the search for matrix **P** that transforms **X** into **Y**, as follows:

Y=PX

Hence, we first calculated the matrix **P** using a matrix **X** that includes all trials and target interval combinations for the visual SCT of our UTND cell population. Using this **P** on other data guarantees that the same transformation is applied to different neural activity sets. Therefore, using the UTND framework we avoided over- or underrepresentation of the information for different target intervals, due to the constant total number of bins across conditions.

### Generating neural trajectories

The TIND information for every trial of all neurons constituted the columns of the **X’** matrix. The principal component coefficients matrix **P** were multiplied by the **X’** matrix to transform the neural data into the space of the original **Y**. Using the same transformation matrix for each trial allowed the comparison of trajectories for different trials and tasks. A locally weighted scatterplot smoothing function was applied to the columns of the **Y** matrix. The first three dimensions of **Y** were used to generate graphical three-dimensional trajectories, while the first eight dimensions, which explained at least 1% of the variance, were used for the other analysis.

### Trajectory radius and variability

The first three PCs explained 10.7%, 3.8%, and 2.3% of the total variance. These three first PCs produced highly stereotyped trajectories with a strong periodicity. In addition, the PC2 and PC3 showed a strong oscillatory structure with a phase difference of π/2 radians during SCT. For these two PCs, we calculated the centroids of the segments of trajectories between adjacent taps. We measured the radius of the 2D trajectory segment as the mean of the Euclidean distances between the centroid and each point in the trajectory segment. The variability of the trajectory was calculated as the standard deviation of the Euclidean distances between the centroid and each point in the trajectory segment across the six serial order elements (three of the SC and three of the CC) for each target interval. Accordingly, the temporal variability of the behavior for each target interval was computed as the standard deviation of the produced intervals within a trial, namely the across-six-serial-order elements of the SCT.

### Neural trajectory decoder

We trained a TDNN [75] to decode the produced intervals from the first PC of the simultaneously recorded neural activity during ST. The TDNN architecture had an input layer with 20 time delays and one hidden 10-unit layer. The output layer consisted of a single unit that was trained to generate a value of 1 when a tap occurred, or 0 otherwise. We trained the network using a Bayesian regularization backpropagation algorithm that minimized the mean squared error of the output. The tap time was defined as the time of the peak of the neural network output higher than a threshold of 0.12. We considered a correctly decoded interval when the decoded and the produced taps times’ difference was less than 60 ms. We used 5-fold cross-validation to evaluate the performance of the neural network.

### dPCA

The dPCA method finds separate decoder (**F**) and encoder (**D**) matrices for each task parameter (∅) by minimizing the loss function,

$$L_{dPCA}=\sum_{\emptyset }{\left\|X_{\emptyset }-F_{\emptyset }D_{\emptyset }X\right\|}^{2}$$

where **X** is a linear decomposition of the data matrix, which contains the instantaneous firing rate of the recorded neurons, into parameter-specific averages:

$$X=\sum_{\emptyset }X_{\emptyset }+X_{noise}$$

The decoder and encoder axes permit us to reduce the data into a few components capturing the majority of the variance of the data dependent on each task parameter [30].

We used the TIND resampled to 30 bins for all target intervals as the input data to the dPCA, and the target interval as the marginalization parameter. Therefore, the length of all the trials for all target intervals was the same. We calculated the bin-by-bin Euclidean distance between the 450-ms first PC and all the target intervals using the PCA and dPCA analyses.

### SVM classifier

We were interested in studying the relation between the neural trajectory dynamics and the instructed interval of the SCT (450 ms, 550 ms, … 1,000 ms). Therefore, we first normalized the length of each segment of the first eight PCs of the neural trajectory associated with a produced interval (the time between two taps) to 30 bins (see inset, Fig 7A). This step was necessary to avoid a bias associated with the length of the segment. Then, we applied a second-layer PCA′ to each of the original neural trajectory segments for each PC independently. We kept the first three PCs, as they explained 96% of the variance. As a result, a point in a new three-dimensional coordinate for each 30-bin trajectory segment was obtained (see Fig 7B). In order to assess which PC had more information about each of the SCT parameters, we carried out a classification procedure for each PC using an SVM algorithm [76]. Each classifier was retrained 10 times, and we used 5-fold cross-validation to evaluate the performance of the classifier. Thus, we identified the PC with more information for each SCT parameter and called it best-PC.

Additionally, we were interested in studying how the size of the neural population used to generate the PCA affected the information contained in the trajectory. We sorted each neuron according to the magnitude of the PCA weights for the best-PC. We iteratively removed the activity of 10% of the neurons with the largest PCA weights for the best-PC until reaching 1% (15 total neurons). Finally, for each population size, we computed the second-layer PCAs on the new trajectories and the corresponding SVM classification.

### Oscillatory activity analysis

To characterize the phase, frequency, and amplitude of the neural trajectories, we calculated a series of nonlinear regression models over the residuals of linear regressions on the first PC projected data. Each inter-tap segment of the projected data was resampled to 30 bins and time normalized to 1 s before calculating the regressions. The general function of the nonlinear models was as follows:

PC=a*sine(2π*t+c)+d

where *t* is time. In addition, the parameter *a* is the amplitude of the oscillatory function, *c* the phase offset, and *d* is a constant. For each trial of both tasks (ST and SRTT), we calculated the MSE.

### Movement kinematics

We applied the Lucas-Kanade optic flow method to measure the monkey’s arm speed during the ST. This method calculates a flow field from the intensity changes between two consecutive video frames. The analyzed video was recorded with a Microsoft Kinect for Windows camera with a 640 × 480 resolution. The optic flow method was applied to a smaller area of 141 × 141 pixels from the original video that contained the monkey’s arm during the whole trial, and no other moving objects. The arm’s movement velocity vector was calculated across all frames as the magnitude of the sum of all the individual flow fields vectors whose magnitude was larger than a predefined threshold. The velocity vector was calculated from the first to the last tap on each correct trial. We reported the speed as the magnitude of the velocity vector. Posteriorly, the kinematic state of the arm was tagged as movement when the velocity vector was larger than a threshold or dwell otherwise. The tagging algorithm considered a change on the kinematic state when the new state lasted longer than three consecutive frames.

### Moving bumps simulations

In order to investigate how the properties of the pattern of neuronal activation affected the generation of population neuronal trajectories, we generated five repetitions of simulations of neuronal activity for each target interval. The individual neuronal activation period was composed of the sum of 20 random gamma functions. The activation period was constant for all the neurons on one simulation, but varied with the target interval: 197-, 205-, 213-, 233-, and 257-ms activation durations for 450-, 550-, 650-, 850-, 1,000-ms target intervals, respectively. The initial activation time for each neuron was adjusted so that the population activation rate followed a Gaussian function as to produce a moving bump pattern. The number of neurons in the simulation was incremented according to the target interval (450 ms, 108 neurons; 550 ms, 120 neurons; 650 ms, 130 neurons; 850 ms, 170 neurons; 1,000 ms, 182 neurons). Fig 11A shows neurons were added randomly in the intermediate portion of the moving bumps.

## Supporting information

S1 Fig Location of the silicon shank for the MPC recordings in Monkey 1 during the ST. MRI cortical surface reconstruction of the macaque brain and the recording position of the Buszaki-64 silicon shank over MPC. The green line corresponds to the anterior-posterior location of the spur of the arcuate sulcus that divides preSMA from SMA. The silicon shank was implanted according to this landmark, so that four more anterior shanks were located in preSMA and other four posterior shanks in SMA. For the recording locations of MPC in Monkeys 1 and 2 during SCT, see Fig 1B of Merchant and colleagues, 2011. AS, arcuate sulcus; CS, central sulcus; IPS, intraparietal sulcus; MPC, medial premotor cortex; preSMA, pre-supplementary motor cortex; PS, principal sulcus; SMA, presupplementary motor cortex proper; ST, synchronization task.
(TIF)

S2 Fig Neural population trajectories during SCT from a subpopulation of cells with task-related activity. The PCA was performed on the time-varying activity of 104 cells that showed at least 15 activation periods on the Poisson-train analysis across the five target durations and six serial order elements of the SCT. The first three PCs explained 32.5% of the total variance. **A.** Projection of the neural activity during the SC and CC of SCT onto the first three PCs. The trajectory completes an oscillatory cycle on every produced interval during the synchronization and continuation phases of the SCT. Target interval in milliseconds is color coded (450, green; 650, blue; 1,000, red). Color progression within each target interval corresponds to the elapsed time. A cube indicates the beginning of each trajectory, while an octahedron indicates the end. **B.** Linear increase of the radii in the oscillatory neural trajectories during SC and CC (mean ± SD, slope = 0.0003, constant = 0.2, *R*^2^ = 0.7, *p* < 0.0001) as a function of target interval. **C**. Linear speed of neural trajectories during SC and CC (mean ± SD, slope = −0.002, constant = 6.3, *R*^2^ = 0.42, *p* = 0.001) as a function of target interval. **D**. Variability of neural trajectories (mean ± SD, normalized data slope = 0.0002, constant = −0.05, *R*^2^ = 0.87, *p* < 0.0001) as a function of target interval. Underlying data are available in https://doid.gin.g-node.org/d315b3db0cee15869b3d9ed164f88cfa/. CC, continuation condition; PC, principal component; PCA, principal component analysis; SC, synchronization condition; SCT, synchronization-continuation task.
(TIF)

S3 Fig Effect of timing and firing rate normalization on the amplitude and speed of neural trajectories. We used different combinations of the time and firing rate normalization of the neural data in order to calculate the PCA coefficients and then the neural trajectories. We fitted a sine function on each of the first 10 PCs and measured their amplitude and speed. For all the possible normalization combinations, we found at least one of the first three PCs that showed a robust fit of the sine function that was accompanied by a monotonic increase in the mean and the variability of the trajectory radius and a similar speed across target intervals. Here, we show only one PC for each normalization combination (see **A, C, E, G**). **(A-F)** These were generated using normalized firing rate data to calculate the trajectories. The left row corresponds to PC radial amplitude and the right row to the PC linear speed. **A,B.** Coefficients computed with time normalized but trajectories calculated on actual time bins, as presented across this paper for SCT. (**A**) PC amplitude increased with target interval: PC3, data slope = 0.00081, constant = 0.011, *R*^2^ = 0.899, *p* < 0.0001, ANOVA main effect target interval, F(4, 20) = 128.69, *p* < 0.0001. (**B**) PC linear speed is similar across target intervals: PC3, nonsignificant linear regression, *R*^2^ = 0.07, *p* = 0.201, ANOVA main effect target interval, F(4, 20) = 22.12, *p* < 0.0001.
**C,D.** Coefficients and trajectories are computed using time-normalized data. (**C**) PC1, data slope = 0.0012, constant = −0.651, *R*^2^ = 0.902, *p* < 0.0001, ANOVA main effect target interval, F(4, 20) = 875.21, *p* < 0.0001. (**D**) PC1, data slope = 0.0048, constant = −1.638, *R*^2^ = 0.98, *p* < 0.0001, ANOVA main effect target interval, F(4, 20) = 390.94, *p* < 0.0001. **E,F**. Coefficients and trajectories are computed using actual time data. (**E**) PC1, data slope = 0.00084, constant = −0.225, *R*^2^ = 0.899, *p* < 0.0001, ANOVA main effect target interval, F(4, 20) = 332.76, *p* < 0.0001. (**F**) PC1, data slope = 0.0034, constant = 0.641, *R*^2^ = 0.686, *p* < 0.0001, ANOVA main effect target interval, F(4, 20) = 100.04, *p* < 0.0001. **G,H**. Same as **(A,B)** but using non-normalized firing rate data to calculate the trajectories. (**G**) PC2, data slope = 0.175, constant = 62.162, *R*^2^ = 0.625, *p* < 0.0001, ANOVA main effect target interval, F(4, 20) = 27.58, *p* < 0.0001. (**H**) PC2, nonsignificant linear regression, *R*^2^ = 0.089, *p* = 0.145, ANOVA main effect target interval, F(4, 20) = 8.18, *p* < 0.001. Underlying data are available in https://doid.gin.g-node.org/d315b3db0cee15869b3d9ed164f88cfa/. PC, principal component; PCA, principal component analysis; SCT, synchronization-continuation task.
(TIF)

S4 Fig State trajectories during ST and SRTT using simultaneously recorded neurons. **A,B.** Three-dimensional neural dynamics trajectory of 650-ms single ST (A) and SRTT (B) intervals. Elapsed time is color coded. The previous and the next taps are marked as red and white spheres, respectively. The stimuli are marked as a white pyramid. Underlying data are available in https://doid.gin.g-node.org/d315b3db0cee15869b3d9ed164f88cfa/. SRTT, serial reaction time task; ST, synchronization task.
(TIF)

S5 Fig State trajectory progress during SCT. **A,B.** One trajectory loop for the second produced interval of the (A) SC and (B) CC, during 450-ms (dark gray) and a 1,000-ms (light gray) target intervals. Trajectory progression marked as colored spheres is as follows: previous tap (green), first inter-tap quarter (cyan), second inter-tap quarter/half interval (blue), third inter-tap quarter (yellow), and next tap (red). Therefore, the neural trajectories follow circular paths with different radii that increase according to the target interval, but with similar speed profiles. Underlying data are available in https://doid.gin.g-node.org/d315b3db0cee15869b3d9ed164f88cfa/. CC, continuation condition; SC, synchronization condition; SCT, synchronization-continuation task.
(TIF)

## Abbreviations

a.u.: arbitrary unit
CC: continuation condition
DAT: Dynamic Attending Theory
dPCA: demixed PCA
EEG: electroencephalogram
MPC: medial premotor cortex
MSE: mean square error
PC: principal component
PCA: principal component analysis
preSMA: pre-supplementary motor area
SC: synchronization condition
SCT: synchronization-continuation task
SI: surprise index
SMA: supplementary motor area
SRTT: serial reaction time task
ST: synchronization task
SVM: support vector machine
TDNN: time-delay neural network
TIND: target interval normalized data
UTND: unit time normalized data
