# Supplementary figures and images for: The amplitude in periodic neural state trajectories underlies the tempo of rhythmic tapping
Source: PLoS Biol. 2019 Apr 8;17(4):e3000054. doi: 10.1371/journal.pbio.3000054 (PMC6472824; doi:10.1371/journal.pbio.3000054)

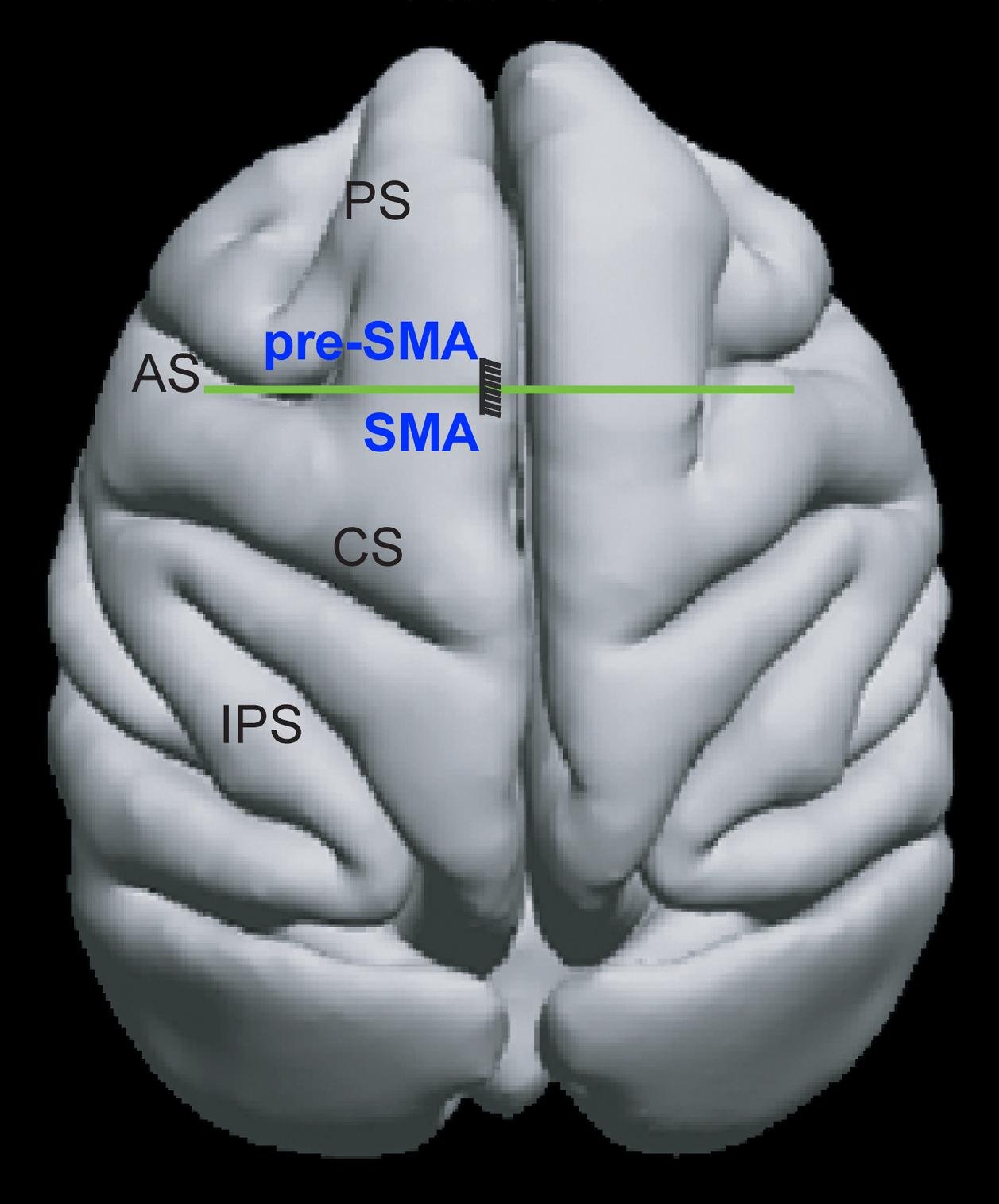

Supplement: S1 Fig — MRI cortical surface reconstruction of the macaque brain and the recording position of the Buszaki-64 silicon shank over MPC. The green line corresponds to the anterior-posterior location of the spur of the arcuate sulcus that divides preSMA from SMA. The silicon shank was implanted according to this landmark, so that four more anterior shanks were located in preSMA and other four posterior shanks in SMA. For the recording locations of MPC in Monkeys 1 and 2 during SCT, see Fig 1B of Merchant and colleagues, 2011. AS, arcuate sulcus; CS, central sulcus; IPS, intraparietal sulcus; MPC, medial premotor cortex; preSMA, pre-supplementary motor cortex; PS, principal sulcus; SMA, presupplementary motor cortex proper; ST, synchronization task. (TIF) [file pbio.3000054.s001.tif]

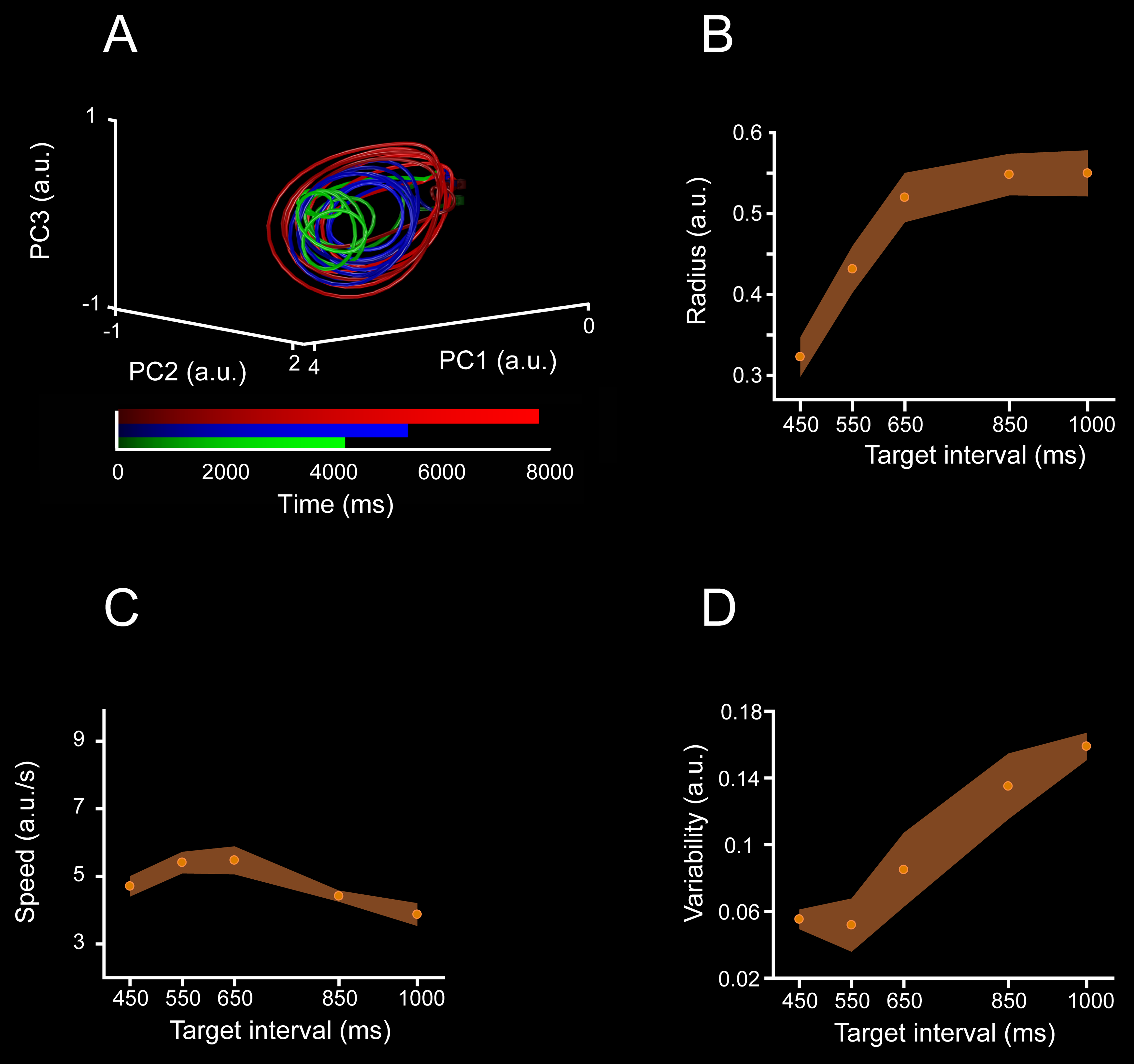

Supplement: S2 Fig — The PCA was performed on the time-varying activity of 104 cells that showed at least 15 activation periods on the Poisson-train analysis across the five target durations and six serial order elements of the SCT. The first three PCs explained 32.5% of the total variance. A. Projection of the neural activity during the SC and CC of SCT onto the first three PCs. The trajectory completes an oscillatory cycle on every produced interval during the synchronization and continuation phases of the SCT. Target interval in milliseconds is color coded (450, green; 650, blue; 1,000, red). Color progression within each target interval corresponds to the elapsed time. A cube indicates the beginning of each trajectory, while an octahedron indicates the end. B. Linear increase of the radii in the oscillatory neural trajectories during SC and CC (mean ± SD, slope = 0.0003, constant = 0.2, R2 = 0.7, p < 0.0001) as a function of target interval. C. Linear speed of neural trajectories during SC and CC (mean ± SD, slope = −0.002, constant = 6.3, R2 = 0.42, p = 0.001) as a function of target interval. D. Variability of neural trajectories (mean ± SD, normalized data slope = 0.0002, constant = −0.05, R2 = 0.87, p < 0.0001) as a function of target interval. Underlying data are available in https://doid.gin.g-node.org/d315b3db0cee15869b3d9ed164f88cfa/. CC, continuation condition; PC, principal component; PCA, principal component analysis; SC, synchronization condition; SCT, synchronization-continuation task. (TIF) [file pbio.3000054.s002.tif]

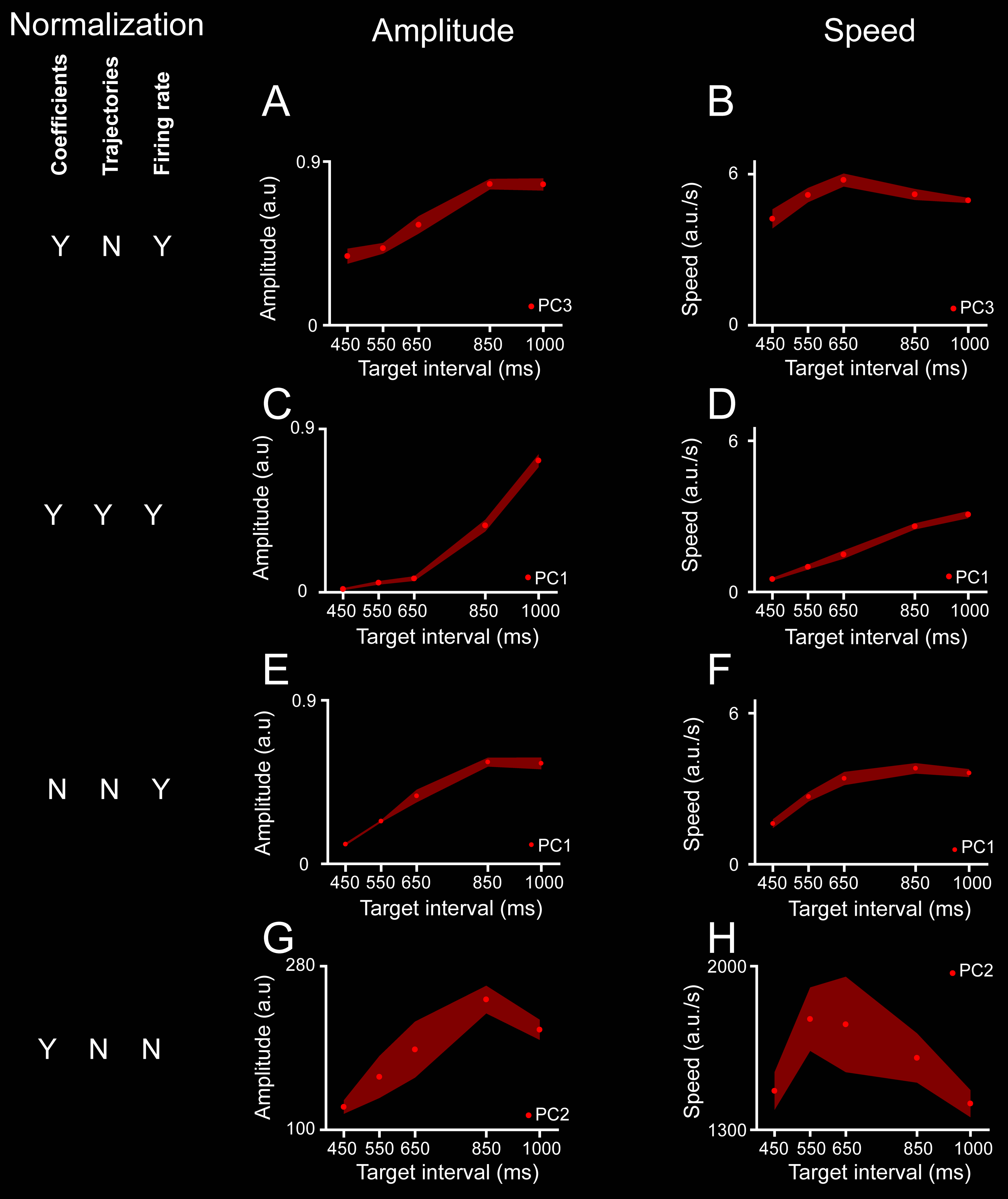

Supplement: S3 Fig — We used different combinations of the time and firing rate normalization of the neural data in order to calculate the PCA coefficients and then the neural trajectories. We fitted a sine function on each of the first 10 PCs and measured their amplitude and speed. For all the possible normalization combinations, we found at least one of the first three PCs that showed a robust fit of the sine function that was accompanied by a monotonic increase in the mean and the variability of the trajectory radius and a similar speed across target intervals. Here, we show only one PC for each normalization combination (see A, C, E, G). (A-F) These were generated using normalized firing rate data to calculate the trajectories. The left row corresponds to PC radial amplitude and the right row to the PC linear speed. A,B. Coefficients computed with time normalized but trajectories calculated on actual time bins, as presented across this paper for SCT. (A) PC amplitude increased with target interval: PC3, data slope = 0.00081, constant = 0.011, R2 = 0.899, p < 0.0001, ANOVA main effect target interval, F(4, 20) = 128.69, p < 0.0001. (B) PC linear speed is similar across target intervals: PC3, nonsignificant linear regression, R2 = 0.07, p = 0.201, ANOVA main effect target interval, F(4, 20) = 22.12, p < 0.0001. C,D. Coefficients and trajectories are computed using time-normalized data. (C) PC1, data slope = 0.0012, constant = −0.651, R2 = 0.902, p < 0.0001, ANOVA main effect target interval, F(4, 20) = 875.21, p < 0.0001. (D) PC1, data slope = 0.0048, constant = −1.638, R2 = 0.98, p < 0.0001, ANOVA main effect target interval, F(4, 20) = 390.94, p < 0.0001. E,F. Coefficients and trajectories are computed using actual time data. (E) PC1, data slope = 0.00084, constant = −0.225, R2 = 0.899, p < 0.0001, ANOVA main effect target interval, F(4, 20) = 332.76, p < 0.0001. (F) PC1, data slope = 0.0034, constant = 0.641, R2 = 0.686, p < 0.0001, ANOVA main effect target interval, F(4, 20) = 100 [file pbio.3000054.s003.tif]

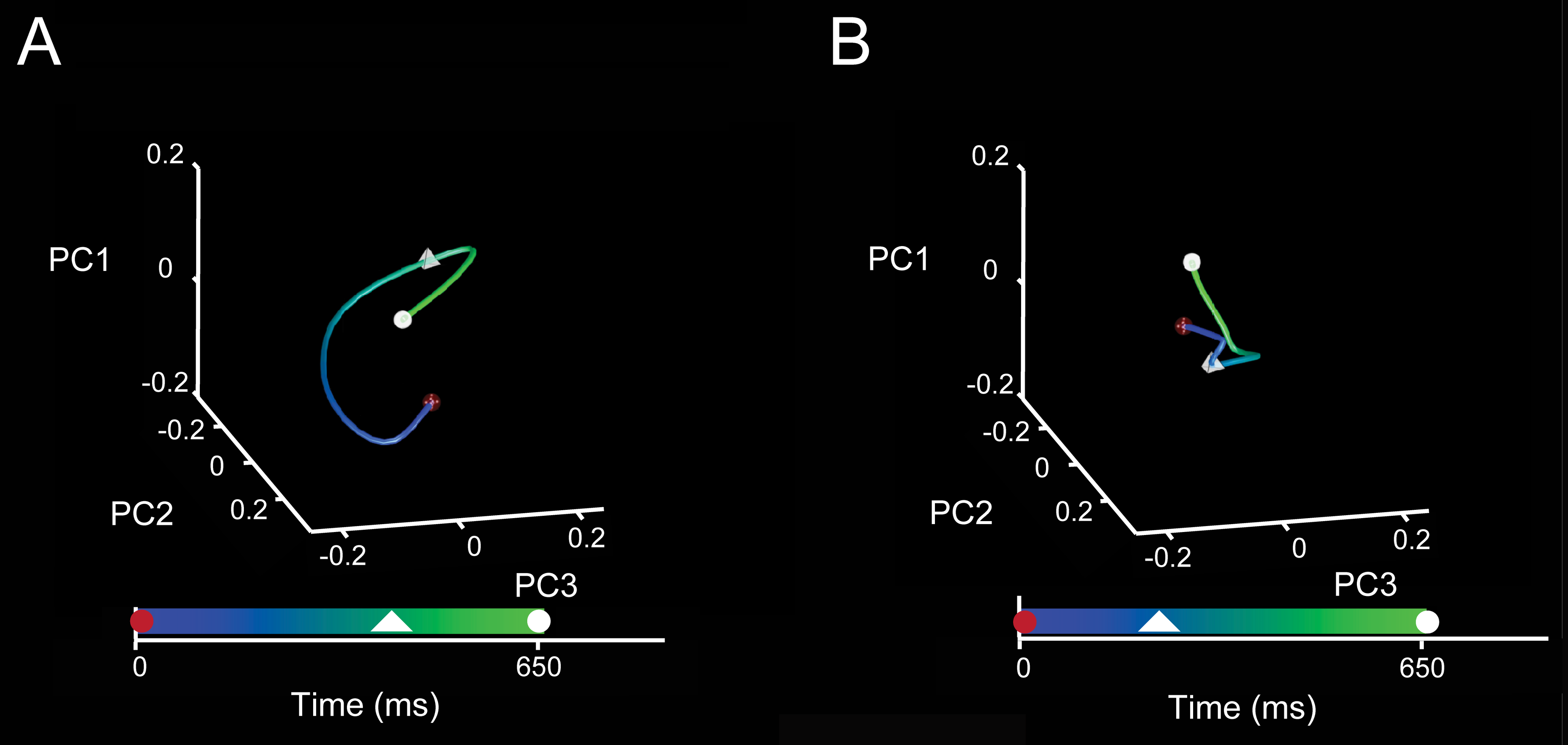

Supplement: S4 Fig — A,B. Three-dimensional neural dynamics trajectory of 650-ms single ST (A) and SRTT (B) intervals. Elapsed time is color coded. The previous and the next taps are marked as red and white spheres, respectively. The stimuli are marked as a white pyramid. Underlying data are available in https://doid.gin.g-node.org/d315b3db0cee15869b3d9ed164f88cfa/. SRTT, serial reaction time task; ST, synchronization task. (TIF) [file pbio.3000054.s004.tif]

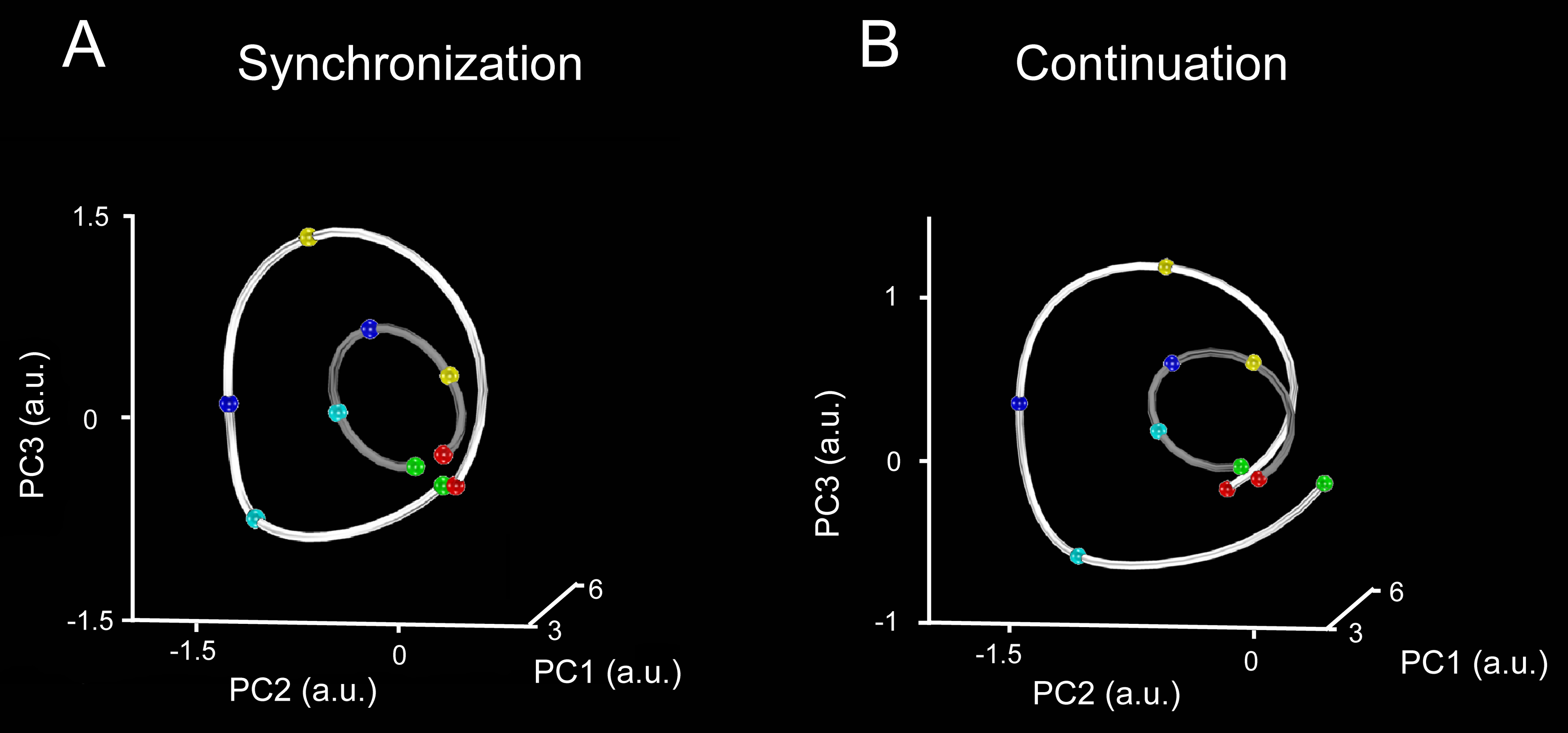

Supplement: S5 Fig — A,B. One trajectory loop for the second produced interval of the (A) SC and (B) CC, during 450-ms (dark gray) and a 1,000-ms (light gray) target intervals. Trajectory progression marked as colored spheres is as follows: previous tap (green), first inter-tap quarter (cyan), second inter-tap quarter/half interval (blue), third inter-tap quarter (yellow), and next tap (red). Therefore, the neural trajectories follow circular paths with different radii that increase according to the target interval, but with similar speed profiles. Underlying data are available in https://doid.gin.g-node.org/d315b3db0cee15869b3d9ed164f88cfa/. CC, continuation condition; SC, synchronization condition; SCT, synchronization-continuation task. (TIF) [file pbio.3000054.s005.tif]
